# Isogenic human stem cell models reveal mutation-dependent protective mechanisms of APOE3 Christchurch

**DOI:** 10.3389/fnagi.2026.1856154

**Published:** 2026-10-01

**Authors:** Carlye Frisch, Lauren Baker, Gayathri Srinivasan, Albert E. Mintah, Salma Leyasi, Morgan Pedroza, Brooke Galyon, David A. Brafman

**Affiliations:** 1 School of Biological and Health Systems Engineering, Arizona State University, Tempe, AZ, United States; 2 School for Engineering of Matter, Transport, and Energy, Arizona State University, Tempe, AZ, United States

**Keywords:** Alzheimer’s disease, amyloid precursor protein processing, APOE3 Christchurch, APP duplication, induced pluripotent stem cells, isogenic models, PSEN1 E280A, tau phosphorylation

## Abstract

**Introduction:**

Alzheimer’s disease (AD) is a progressive neurodegenerative disorder influenced by both causal mutations and genetic modifiers. The rare APOE3 Christchurch (APOE3ch) variant has been associated with delayed disease onset in a familial Alzheimer’s disease case, suggesting a potential protective role. However, the mechanisms underlying this protection, particularly across different genetic backgrounds, remain unclear.

**Methods:**

We generated isogenic human induced pluripotent stem cell (hiPSC)–derived neural cultures harboring either PSEN1 E280A or amyloid precursor protein duplication (APPdp) mutations, with and without the APOE3ch variant, using prime editing. Neural differentiation, amyloid-beta production, amyloid precursor protein processing, tau phosphorylation and transcriptomic profiles were assessed.

**Results:**

APOE3ch did not affect neural differentiation or apolipoprotein E secretion. However, it exhibited mutation-dependent effects on Alzheimer’s disease phenotypes. In amyloid precursor protein duplication cultures, APOE3ch significantly reduced amyloid-beta production and shifted processing toward non-amyloidogenic pathways, accompanied by decreased phosphorylated tau. In contrast, in PSEN1 E280A cultures, APOE3ch had minimal impact on amyloid-beta levels but increased non-amyloidogenic processing and altered total tau without affecting phosphorylation. Transcriptomic analysis revealed both shared and mutation-specific effects, with consistent modulation of pathways related to synaptic function, including Wnt and cadherin signaling.

**Discussion:**

These findings demonstrate that APOE3ch acts as a context-dependent modifier of Alzheimer’s disease pathology, with protective effects varying by genetic background. The variant consistently influences pathways associated with synaptic resilience while differentially modulating amyloid and tau pathology. This work provides mechanistic insight into APOE3ch-mediated protection and highlights the importance of genetic context in developing targeted therapeutic strategies for Alzheimer’s disease.

## 1 Introduction

Alzheimer’s disease (AD) is a progressive neurodegenerative disorder characterized by cognitive decline, memory loss, and widespread neuropathological changes, affecting millions worldwide (Holtzman et al., 2011). While most cases are sporadic (SAD), a small subset is caused by autosomal-dominant AD (ADAD) mutations in presenilin 1 (PSEN1), presenilin 2 (PSEN2), and amyloid precursor protein (APP), leading to early-onset AD with hallmark amyloid plaques and neurofibrillary tangles (Bateman et al., 2011; Bird, 2008; Selkoe and Hardy, 2016). Understanding how genetic factors modify AD pathology is essential for developing targeted therapies.

In addition to ADAD mutations, various genetic risk variants influence AD susceptibility. Among these, the APOE gene plays a central role in modulating disease risk, primarily through its involvement in lipid transport, synaptic function, and amyloid-beta metabolism in the brain (Martens et al., 2022; Raulin et al., 2022). The three primary APOE isoforms (APOE2, APOE3, and APOE4) have distinct effects on AD onset and age-related progression, with APOE4 increasing susceptibility and APOE2 offering protection (Corder et al., 1993; Saunders et al., 1993; Wolf et al., 2013). Studying protective forms of disease, specifically, APOE variants with potential resilience against AD pathology, is crucial to identify strategies to mitigate disease onset and progression. Recently, a rare variant, APOE3 Christchurch (APOE3ch), was identified in an individual who was heterozygous for the pathogenic PSEN1 E280A mutation and homozygous for APOE3ch and who exhibited a markedly delayed onset of disease (Arboleda-Velasquez et al., 2019). This suggests that APOE3ch may confer resilience against AD pathology, though the mechanisms underlying its protective effects remain poorly understood. Early findings indicate that APOE3ch may disrupt interactions with heparan sulfate proteoglycans (HSPGs), which influence amyloid aggregation and tau pathology (Arboleda-Velasquez et al., 2019). Additionally, emerging evidence suggests that APOE3ch may modulate neuroinflammatory responses and enhance synaptic resilience, though these mechanisms remain speculative (Chen et al., 2024; Nelson et al., 2023).

Notably, most initial studies of APOE3ch have focused on its impact on tau-related phenotypes due to early clinical evidence indicating a strong effect of this pathology (Arboleda-Velasquez et al., 2019; Marino et al., 2024; Nelson et al., 2023). However, it remains unclear whether APOE3ch influences APP processing and amyloid-beta metabolism, key factors in AD pathogenesis. Furthermore, it is unknown whether APOE3ch’s protective effects extend beyond PSEN1*^E280A^* carriers or if it can mitigate disease phenotypes across other ADAD mutations. Addressing these gaps is critical for determining the broader therapeutic potential of APOhE3ch in AD.

To address these questions, we generated isogenic human induced pluripotent stem cell (hiPSC)-derived neural culture models with and without the APOE3ch variant. Specifically, using a highly efficient prime editing system (Frisch et al., 2023), we introduced APOE3ch into hiPSC lines carrying PS1*^E280A^* and APP duplication (APPdp) mutations. In turn, we differentiated these cells into neural cultures and assessed key AD-related phenotypes, including amyloid-beta (Aβ) production, APP processing, and tau phosphorylation, revealing mutation-dependent effects of APOE3ch. Transcriptomic analysis identified significant changes in AD-related pathways, particularly Wnt and cadherin signaling. Overall, by utilizing a well-controlled isogenic model, this study provides valuable insights into the broader protective mechanisms of the APOE3ch variant in the context of Alzheimer’s disease.

## 2 Materials and methods

### 2.1 hiPSC culture

Human induced pluripotent stem cells were cultured on feeder-free Cultrex Basement Membrane Extract (RandD Systems) and maintained in mTeSR™ Plus medium (STEMCELL Technologies). Cells were passaged every 3–4 days or at 70% confluency using Accutase (ThermoFisher). Upon plating, hiPSCs were suspended in mTeSR™ Plus medium supplemented with 5 μM Y-27632 ROCK inhibitor (Tocris). Cells were routinely tested for with the MycoAlert PLUS kit (Lonza) and the Lucetta™ Luminometer (Lonza).

### 2.2 APP*^dp^* APOE3ch hiPSC isogenic cell line generation

Amyloid precursor protein duplication hiPSCs (Israel et al., 2012) were passaged onto Cultrex Basement Membrane Extract-coated 12-well plates in mTeSR™ Plus medium and 5 μM Y-27632 ROCK inhibitor. After 24 h, cells at 60% confluency were transfected. Prior to transfection, plates were washed with PBS, and media was replaced. Transfection was performed using APOE3ch targeting PE3b epegRNA (Supplementary Table 1) and PEmax prime editor (PE; Chen et al., 2021). A total of 2.25 ng plasmid DNA was added, maintaining a 1:1:6 ratio of pegRNA:BFP:PE, along with 8 μL of Lipofectamine™ Stem Transfection Reagent (ThermoFisher) and 50 μL of OptiMEM™ with GlutaMAX (ThermoFisher) per well. After 24 h, cells were washed with PBS, and media was replaced and 48 h post-transfection, the cells were the cells were treated with Accutase and incubated at 37°C for 5 min, then spun at 200 g for 5 min. Cells were resuspended in mTeSR™ Plus medium containing 1X CloneR™ (STEMCELL Technologies) and 5 μM Y-27632. GFP-positive cells were sorted using a 100 μm nozzle on the BD FACSymphony™ S6 Cell Sorter (BD Biosciences) into Cultrex-coated 96-well plates. Media was changed to mTeSR™ Plus every other day for the next 10 days. Once clones reached 50% confluency, they were passaged using established protocols into Cultrex-coated 12-well plates for further analysis and banking.

### 2.3 PS1*^E280A^* APOE3ch iPSC isogenic cell line generation

Non-demented control hiPSCs (Brookhouser et al., 2021) were passaged onto Cultrex Basement Membrane Extract-coated 12-well plates with mTeSR™ Plus medium and 5 μM Y-27632. After 24 h, cells at 60% confluency were transfected. Transfection was carried out with a PS1E280A targeting PE3 epegRNA (Supplementary Table 1), PEmax prime editor (PE), and a vector expressing MLH1dn. A total of 1.375 ng plasmid DNA was used with a 1:1:3:6 ratio of pegRNA:BFP:MLH1dn:PE, 6 μL of Lipofectamine™ Stem Transfection Reagent (ThermoFisher), and 50 μL of OptiMEM™ with GlutaMAX (ThermoFisher) per well. Post-transfection, cells were processed as described above. To introduce the APOE3ch mutation, the heterozygous PS1E280A iPSC clone was transfected with APOE3ch targeting PE3b epegRNA (Supplementary Table 1) and PEmax prime editor. A total of 1 ng plasmid DNA was used with a 1:1:6 ratio of pegRNA:BFP:PE, following similar protocols.

### 2.4 Genotyping and sequence analysis

Genotyping and sequencing followed protocols outlined in previous publications (Frisch et al., 2023) using Phusion^®^ High-Fidelity DNA Polymerase PCR. PCR primers are provided in Supplementary Table 2.

### 2.5 Off-target analysis

Off-target analysis for PS1*^E280A^* and APOE3ch was conducted using protocols outlined in previous publications (Frisch et al., 2023), identifying the top 3 off-target sites. PCR primers for these off-target sites are in Supplementary Table 2.

### 2.6 Tri-lineage differentiation of iPSCs

Differentiation protocols for iPSC clones can be found in previous publications (Frisch et al., 2023).

### 2.7 Karyotype analysis

Karyotype analysis was conducted for each clonal line by Cell Line Genetics, utilizing standard procedures for G-banding as we have previously described (Frisch et al., 2023).

### 2.8 Immunofluorescence

For immunofluorescence of hiPSCs, cells were washed with PBS, fixed with BD Cytofix Fixation Buffer (BD Biosciences), and permeabilized with BD Phosflow Perm Buffer III (BD Biosciences). Following overnight incubation with primary antibodies (diluted in Stain Buffer, FBS), secondary antibodies were applied for 1 h at room temperature. Nuclei were stained with Hoechst, and cells were stored in PBS for imaging. Antibodies and dilutions are provided in Supplementary Table 3. Fluorescence images were analyzed using identical image acquisition and processing parameters across all samples. Brightness and contrast settings were maintained consistently for each fluorescence channel to enable unbiased comparisons between experimental groups. For each image, mean fluorescence intensity (MFI) was quantified, and background fluorescence was measured from a region of interest lacking detectable cellular signal. Background-corrected fluorescence intensity was calculated by subtracting the background MFI from the measured MFI for each marker. To account for differences in cell number, background-corrected fluorescence intensities were normalized to the corresponding Hoechst fluorescence intensity. Statistical analyses were performed using GraphPad Prism. Data normality was assessed using the Shapiro–Wilk test, and all datasets satisfied the assumption of normality. Comparisons between APOE3 and APOE3ch cultures were performed using an unpaired two-tailed Welch’s *t*-test. Statistical significance was defined as *P* < 0.05.

### 2.9 Microscopy

Imaging was performed on a Nikon Ti2-Eclipse inverted microscope with a Lumencor SOLA SE Light Engine and Semrock bandpass filters.

### 2.10 Flow cytometry

Human induced pluripotent stem cells were washed once with PBS and incubated in Accutase for 5 min at 37 °C to facilitate detachment. Accutase was neutralized using a 1:1 ratio of Accutase to DMEM, and cells were centrifuged at 200*g* for 5 min. The resulting cell pellet was resuspended in PBS, and 5 μL of antibodies (SSEA4 and Tra-1-81, along with isotype control antibodies) were added to each sample (Supplementary Table 3). Samples were incubated on ice for 1 h under light-protected conditions. Following incubation, cells were washed twice with PBS, resuspended in PBS, and analyzed using the ThermoFisher Attune NxT flow cytometer. Gating was established using isotype controls, and data analysis was performed using FlowJo software.

### 2.11 Human neural progenitor cell (hNPC) generation

Induced pluripotent stem cells (iPSCs) were differentiated into human neural progenitor cells (hNPCs) using STEMCELL Technologies’ SMADi Neural Induction Kit, following the manufacturer’s embryoid body (EB) protocol for neural differentiation until passage 1 (day 17–19) (Knittel et al., 2022). At passage 1, hNPCs were incubated with Accutase for 5 min at 37 °C to facilitate detachment. The cells were then lifted from the well, and Accutase was neutralized with DMEM at a 1:1 ratio. The cell suspension was centrifuged at 300*g* for 5 min, and the resulting pellet was resuspended in neural expansion media as we previously described (Knittel et al., 2022). Cells were seeded at a density designed to reach ~60% confluency every 4–5 days. NPCs were expanded in neural expansion media and cryopreserved between passages 1 and 7 at a density of 2 million cells/mL in cryopreservation media (90% fetal bovine serum, 10% dimethyl sulfoxide). Cryopreserved hNPCs were stored at −150 °C until further differentiation.

### 2.12 Neural differentiation of hNPCs

HNPCs were differentiated into neurons following previously published protocols (Knittel et al., 2022), with modifications to enhance cell attachment and differentiation efficiency. Corning^®^ Enhanced Attachment Microcarriers (MCs) were coated with 15 μg/mL poly-L-ornithine (PLO) and incubated overnight at 4 °C. Following PLO coating, MCs were washed twice with PBS and once with DMEM/F12 (Life Technologies) before being coated with 20 μg/mL Laminin diluted in DMEM/F12. Laminin coating was incubated overnight at 4 °C. The Laminin solution was then removed, and MCs were suspended in neural expansion media before seeding hNPCs. With these modifications, hNPCs were differentiated into neurons according to Knittel et al. (2022) for 30–35 days. Upon completion of differentiation, cells were dissociated and plated onto 24-well Cultrex-coated plates for an additional 7–10 days prior to analysis. Neuronal cultures were fixed between days 7–10 post-dissociation from MCs. 6-day conditioned media and RIPA lysates were collected on day 10 post-dissociation for further analysis.

### 2.13 Protein quantification from cell lysates

Total cellular protein levels were measured using the Pierce BCA Protein Assay Kit (ThermoFisher) according to the manufacturer’s protocols. Total tau levels in neural cultures were quantified using the Human Tau (Total) ELISA Kit (ThermoFisher) and normalized to total protein content. Phosphorylated tau (pT231) was measured using the Human Tau (Phospho) [pT231] ELISA Kit (ThermoFisher) and similarly normalized to total protein levels. Total APP levels were determined using Human Amyloid Precursor Protein ELISA Kits (ThermoFisher) and normalized to total cellular protein. β-CTF levels were measured with the IBL APP β-CTF ELISA Kit. All ELISAs were performed following manufacturer instructions.

### 2.14 Protein quantification from conditioned media

Neurons were seeded in 24-well Cultrex-coated plates at 1 million cells per well. On day 4 post-dissociation, a full media change with neural differentiation media (Knittel et al., 2022) initiated conditioned media collection. On day 10 post-dissociation, conditioned media was harvested and centrifuged at 200 × *g* for 5 min at room temperature. The clarified supernatant was transferred to new tubes for analysis. APOE levels were measured from 6-day conditioned media using an APOE ELISA Kit (ThermoFisher) and normalized to total cellular protein. Amyloid-β40 and Amyloid-β42 concentrations were quantified using RandD Systems Human Aβ(40) and Aβ(42) ELISA kits and normalized to total protein levels. Soluble APP-α and soluble APP-β were measured from 6-day conditioned media using IBL ELISA kits following manufacturer protocols. Soluble APP levels were normalized to total APP levels measured in the matched neural cultures.

### 2.15 RNA isolation and sequencing

RNA was isolated from neural cultures between days 7–10 post-dissociation using previously established protocols with minor modifications (29775730). Cells were lysed in cold TRIzol™ Reagent (ThermoFisher) and incubated at 65 °C for 5 min. Chloroform (200 μL per 1 mL TRIzol) was added, samples were vortexed, incubated on ice for 5 min, and centrifuged at 10,000 × *g* for 5 min at 4 °C. The aqueous phase was collected, mixed 1:1 with 70% ethanol, and RNA was purified using the NucleoSpin RNA Kit (Macherey-Nagel). RNA sequencing was performed by BGI Americas using the DNBseq platform with paired-end 150 bp reads. Differential expression analysis was performed using DESeq2 (Love et al., 2014). Gene ontology and pathway analyses were conducted using the PANTHER database v19.0 with Fisher’s Exact Test and FDR correction. Upstream regulator analysis was performed using Ingenuity Pathway Analysis (IPA; QIAGEN) using full gene datasets from each isogenic comparison. Significant upstream regulators were defined by a Benjamini-Hochberg-adjusted *P*-value ≤ 0.05 and an absolute IPA activation z-score ≥2, with positive and negative z-scores indicating predicted activation and inhibition, respectively. To identify upstream regulators specifically associated with Alzheimer’s disease, significant IPA regulators were further compared with the AD-associated transcriptional regulators and signaling molecules compiled by Chen et al. (2013). The RNA-seq datasets generated in this study have been deposited in the NCBI Gene Expression Omnibus (GEO) under accession number GSE338592.

### 2.16 Statistical analysis

Statistical significance between groups was determined using Brown-Forsythe and Welch ANOVA tests for multiple comparisons, or Welch’s *t*-test for comparisons between groups with unequal variances. A *p*-value < 0.05 was considered statistically significant. All analyses were performed using GraphPad Prism version 8.4.1. Data are presented as mean ± SEM unless otherwise specified.

## 3 Results

### 3.1 Generation of an isogenic hiPSC-derived Alzheimer’s disease model to study the impact of APOE3ch variant

To investigate the potential mechanisms by which the APOE3ch variant modulates AD-related phenotypes and processes, we sought to generate isogenic hiPSC lines carrying the APOE3ch variant using a highly efficient prime editing method that we previously developed (Frisch et al., 2023). Because the original case report examined the APOEch variant in the context of a PS1*^E280A^* carrier, we first engineered a hiPSC line with this mutation. To that end, we used our gene editing strategy to introduce the PS1*^E280A^* mutation into a previously characterized healthy, non-demented control (NDC) hiPSC line with an APOE 3/3 genotype (Brookhouser et al., 2021). One clone was selected with the desired PS1*^E280A^* mutation (Figure 1a) and no off-target editing (Supplementary Figure 1). Next, we used the same gene editing approach to introduce the APOEch variant into the PS1*^E280A^* line. Genotyping at the APOE^112^, APOE^136^, and APOE^158^ site revealed several clones with no off-targeting editing (Supplementary Figure 2) that were homozygous for the APOE3ch mutation (Figure 1a and Supplementary Figure 3a). We selected 3 clones for further analysis to minimize the possibility that observed phenotypes were a result of clonal variation.

**FIGURE 1:**
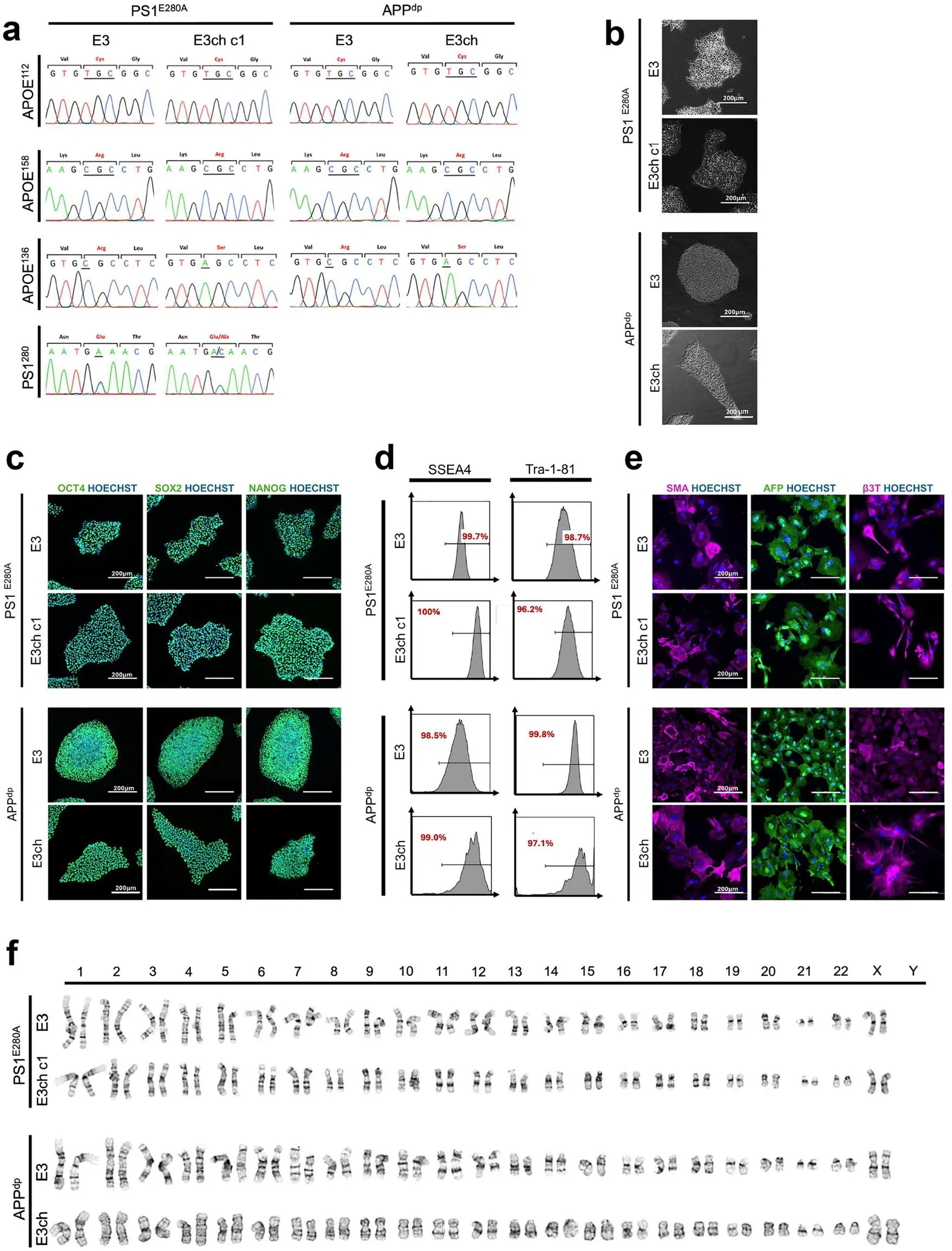
Generation and validation of isogenic hiPSC lines carrying APOE3 Christchurch (APOE3ch) in familial Alzheimer’s disease genetic backgrounds. **(a)** Sanger sequencing chromatograms confirming introduction of the PSEN1 E280A (PS1^E280A^) mutation and generation of homozygous APOE3ch clones in PS1^E280A^ and APP duplication (APP^dp^) hiPSC lines. Editing was confirmed at APOE codons 112, 136, and 158. **(b)** Representative phase-contrast images demonstrating typical hiPSC colony morphology across parental and edited clones. **(c)** Immunofluorescence staining showing expression of pluripotency markers OCT4, SOX2, and NANOG across parental and edited hiPSC lines. **(d)** Flow cytometry analysis demonstrating high expression of pluripotency-associated surface markers TRA-1-81 and SSEA4. **(e)** Trilineage differentiation assays demonstrating differentiation potential into ectoderm, mesoderm, and endoderm lineages. **(f)** G-banded karyotype analysis confirming normal euploid chromosomal structure across all generated clones.

Since the original case report only examined the ApoEch in the context of PS1*^E280A^* carriers, it is unclear the extent to which AD-related phenotypes are reproduced across additional familial AD (FAD) mutations. As such, we also aimed to generate additional isogenic hiPSC lines in the context of another familial Alzheimer’s disease (FAD) mutation in APP caused by a duplication of APP (APP*^dp^*). Critically, previous studies in hiPSCs with this APP mutation demonstrated the presence of robust AD-related phenotypes when differentiated to neural cell types (Israel et al., 2012). Thus, since we have previously shown that introduction of other APOE variants into this APP*^DP^* hiPSC line modulates disease-related phenotypes (Brookhouser et al., 2021), we speculated that modulation of these phenotypes by introduction of the APOEch variant would readily be observed. Genotyping at the APOE^112^, APOE^136^, and APOE^158^ site confirmed one homozygous APOE3ch clones (Figure 1a), with no off-target edits detected (Supplementary Figure 4).

Comprehensive characterization of all edited clones included assessments of stem cell-like morphology, pluripotency marker expression, differentiation capability, and karyotypic stability. Each clone displayed characteristic hiPSC morphology (Figure 1b and Supplementary Figure 3b), expressed markers of the undifferentiated state (OCT4, SOX2, NANOG, TRA-1-81, SSEA4) (Figures 1c, d and Supplementary Figures 3c, d), and demonstrated trilineage differentiation potential with expression of mesoderm, endoderm, and ectoderm markers (Figure 1e and Supplementary Figure 3e). Finally, karyotype analysis confirmed a normal euploid karyotype across all lines (Figure 1f and Supplementary Figure 3f). Overall, we have successfully generated and characterized two sets of isogenic APOE3 and APOE3ch hiPSC lines, each containing distinct familial mutations, and exhibiting all the hallmark traits of hiPSCs.

### 3.2 APOE3ch does not affect neural differentiation of hiPSCs

Next, we employed scalable microcarrier (MC)-based differentiation protocols developed in our lab (Knittel et al., 2022) to generate neural cultures from each isogenic pair. Human neural progenitor cells (hNPCs) were first derived using established methods with modifications (Knittel et al., 2022), then seeded onto poly-L-ornathine/laminin (LN)-coated microcarriers in 6-well ultra-low attachment plates. The medium was switched to neural differentiation medium, and after at least 30 days, cultures were dissociated and replated onto Matrigel-coated tissue culture plates.

Analysis of neural cultures generated from the PS1*^E280A^* and APP*^DP^* isogenic cell lines revealed a large percentage of cells that expressed the mature neuronal markers TUJ1, MAP2, and NEUN (Figure 2a). In addition, these differentiated cultures also resulted in the generation of numerous S100β-positive astrocytes (Figure 2b). This is significant because astrocytes are the primary source of APOE production in the brain, and disease-related phenotypes are influenced by the interactions between astrocyte-secreted APOE and neurons (Huang, 2010; Huang and Mucke, 2012). To quantitatively determine whether APOE3ch altered neuronal or astrocytic differentiation, we quantified immunofluorescence staining for these lineage markers. No significant differences were observed between APOE3 and APOE3ch cultures in the expression of B3T, MAP2, GFAP, or S100β in either the PS1*^E280A^* or APP*^dp^* background (Supplementary Figure 6), indicating comparable neuronal and astrocytic differentiation across the isogenic cultures.

**FIGURE 2:**
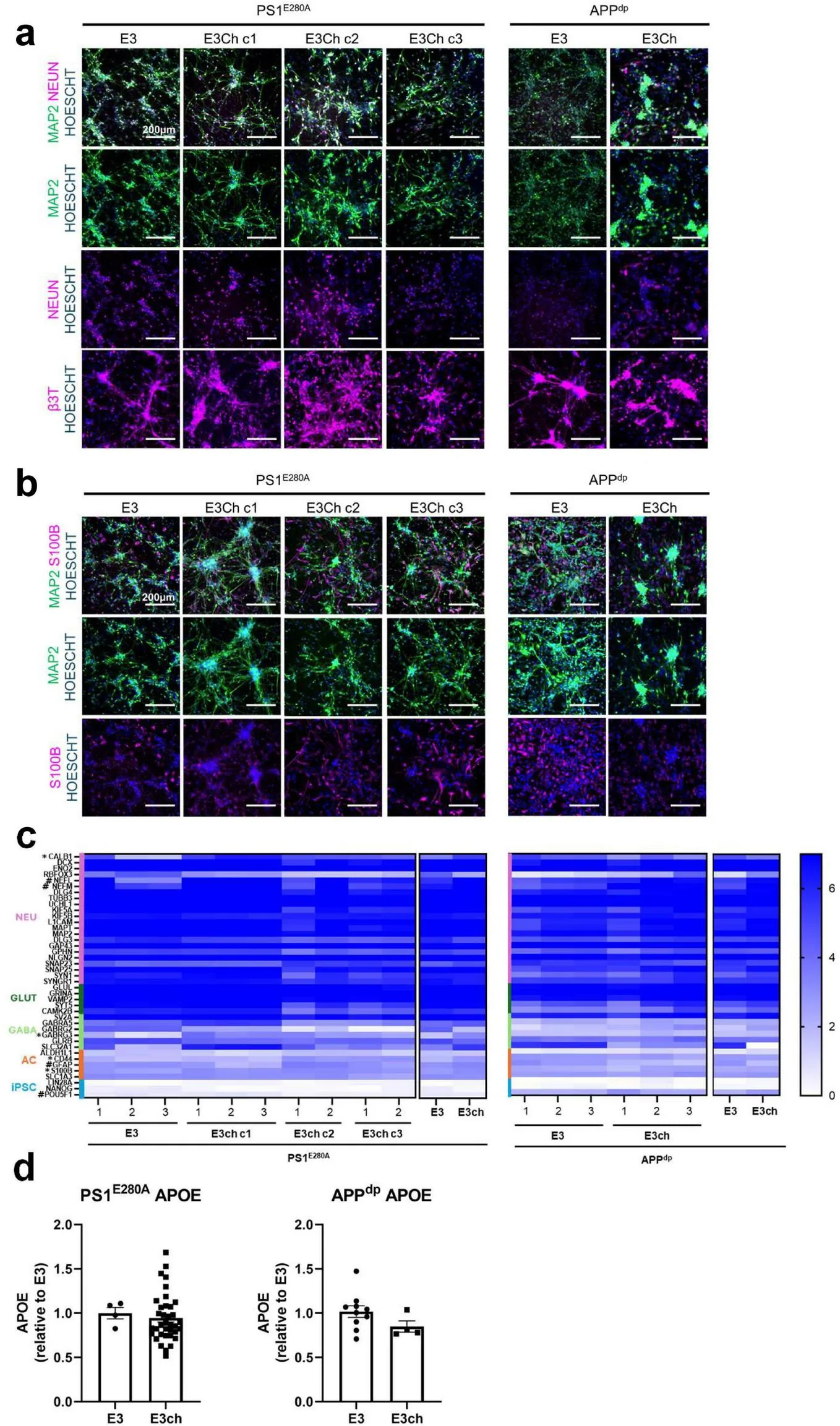
Characterization of neural differentiation and APOE secretion in APOE3 and APOE3ch isogenic hiPSC-derived cultures across familial Alzheimer’s disease backgrounds. **(a)** Representative immunofluorescence images of differentiated neural cultures derived from PSEN1 E280A and APP duplication (APPdp) APOE3 and APOE3ch clones showing expression of mature neuronal markers MAP2, NEUN, and βIII-tubulin (TUJ1). **(b)** Representative immunofluorescence images demonstrating the presence of astrocytes and neurons across all clones, identified by S100β and MAP2 expression, respectively. **(c)** RNA-seq–based gene expression analysis of neural cultures generated across multiple independent differentiations for all clones used in this study. Comparable expression levels of neuronal subtype markers and astrocytic markers were observed between APOE3 and APOE3ch cultures. Statistically significant differences between APOE3 and APOE3ch are denoted by an asterisk (*) for PS1^E280A^ cultures and a number sign (#) for APP^dp^ cultures **(d)** Quantification of secreted APOE levels measured by ELISA from conditioned media of differentiated neural cultures. For RNA-seq data, each column represents an independent well from independent differentiations. For ELISA data, replicates included PS1^E280A^ APOE3 (*n* = 4; 1 independent wells from differentiation #1, 1 independent well from differentiation #2, 2 independent wells from differentiation #3), PS1^E280A^ APOE3ch (*n* = 36; Clone 1: 6 independent wells from differentiation #1, 6 independent wells from differentiation #2, 8 independent wells from differentiation #3; Clone 2: 6 independent wells from differentiation #1, 1 independent well from differentiation #2, 2 independent wells from differentiation #3; Clone 3: 3 independent wells from differentiation #1, 2 independent wells from differentiation #2, 2 independent wells from differentiation #3), APP^dp^ APOE3 (*n* = 10; 1 independent well from differentiation #1, 6 independent wells from differentiation #2, 3 independent wells from differentiation #3), and APP^dp^ APOE3ch (*n* = 4; 2 independent wells from differentiation #1, 2 independent wells from differentiation #2).

To further assess whether the APOE3ch variant influenced neural differentiation, we analyzed RNA-seq data from differentiated cultures generated across multiple independent differentiations. The analysis of global correlation coefficients demonstrated a high degree of reproducibility between independent differentiations in both the PS1*^E280A^* (Supplementary Figure 5a) and APP*^DP^* (Supplementary Figure 5b) isogenic sets. Consistent with the immunofluorescence quantification, APOE3 and APOE3ch cultures exhibited comparable expression levels of genes associated with mature neurons (e.g., *DCX, MAP2, MAPT, NEFL, NEFM, TUBB3*) as well as the presence of functional synapses (e.g., *SNAP25, SYN1*) (magenta cluster in Figure 2c).

Previous studies with mouse (Andrews-Zwilling et al., 2010) and hiPSC-based (Wang et al., 2018) systems have shown that APOE genotypes might influence GABAergic interneuron generation and survival. Analysis of gene expression profiles associated with excitatory glutamatergic (dark green cluster in Figure 2c) and GABAergic neurons (light green cluster in Figure 2c) revealed that there were similar levels between APOE3 and APOEch cultures in the relative expression of the neuronal subtype-specific markers. Additionally, APOE3 and APOEch cultures expressed similar levels of astrocytic markers (orange cluster in Figure 2c), indicating that the APOEch variant had no effect on astrocyte generation from hiPSCs. Together, the immunofluorescence and gene expression analyses provide complementary evidence that APOE3ch does not substantially alter the neuronal or astrocytic composition of the differentiated cultures.

Research in animal models (Bien-Ly et al., 2012) and hiPSC-based models (Wang et al., 2018) has indicated that the APOE3 genotype leads to higher total APOE levels compared to the APOE4 genotype. To explore this further, we measured the amount of secreted APOE in the medium of differentiated cultures. In both the PS1*^E280A^* and APP*^dp^* lines (Figure 2d), APOE3 and APOE3ch cultures secreted similar levels of APOE into the extracellular medium, suggesting that the introduction of the APOE3ch variant did not impact APOE production. Collectively, these findings demonstrate that APOE3ch does not significantly alter neuronal differentiation, astrocyte generation, neural cell composition, or APOE secretion in either the PS1*^E280A^* or APP*^dp^* isogenic background.

### 3.3 APOE3ch reduces Aβ in APP*^dp^* but not PS1*^E280A^* cultures

In the central nervous system, neurons are the primary source of extracellular Aβ, which is generated through amyloidogenic processing of amyloid precursor protein (APP), while non-neuronal cells, particularly astrocytes, play important roles in Aβ uptake and clearance from the extracellular space (Liu et al., 2013). Accordingly, extracellular Aβ accumulation reflects the balance between APP processing and Aβ production, trafficking, uptake, degradation, and clearance, rather than production alone. Increased Aβ production can contribute substantially to extracellular Aβ accumulation and the amyloid cascade underlying Alzheimer’s disease pathology (Chow et al., 2010; Zhang et al., 2011) but impaired turnover and clearance can also promote Aβ accumulation. Indeed, APOE has been shown to regulate extracellular Aβ metabolism independently of Aβ synthesis, and APOE isoforms differentially influence the clearance of soluble Aβ from the brain (Castellano et al., 2011; DeMattos et al., 2004).

These processes are also mechanistically interconnected. Receptors such as LRP1 can regulate APP endocytosis and amyloidogenic processing while also mediating cellular uptake and clearance of extracellular Aβ (Kanekiyo and Bu, 2014; Kanekiyo et al., 2013; Van Gool et al., 2019). As such, APOE-mediated effects on receptor-dependent trafficking may influence both APP biology and downstream Aβ turnover, making it difficult to separate effects on production from effects on clearance. Consistent with this broader role, APOE isoforms have been shown to differentially regulate multiple aspects of Aβ metabolism, including APP trafficking and processing, Aβ production, cellular uptake, and clearance, with APOE4 linked to increased APP endocytosis and Aβ production compared with APOE2 and APOE3 (He et al., 2007; Ye et al., 2005). Similarly, our work with isogenic hiPSCs has shown that, compared with APOE3, APOE4 increases and APOE2 decreases levels of secreted Aβ (Brookhouser et al., 2021; Zhao et al., 2020). Regarding the APOE3ch variant, early *in vitro* studies indicated that APOE3ch reduces Aβ42 aggregation compared with wild-type APOE3 (Arboleda-Velasquez et al., 2019). The original case report further suggested that the PS1*^E280A^* individual would likely have exhibited a substantially greater amyloid burden in the absence of the protective APOE3ch genotype (Arboleda-Velasquez et al., 2019). However, it remains unclear whether the amyloid-modifying effects of APOE3ch are mediated primarily through altered APP trafficking and processing, Aβ aggregation, uptake and clearance, or a combination of these interconnected mechanisms.

To determine the extent to which Aβ production is influenced by APOEch, we measured Aβ40 and Aβ42 levels released into the extracellular medium from neural cultures using ELISA. In the PS1*^E280A^* isogenic pair, no significant differences in Aβ levels were observed between the APOE3 and APOE3ch cell lines, indicating that the APOE3ch variant does not affect Aβ production in the context of the PS1*^E280A^* mutation (Figures 3a, b). However, in the APP*^dp^* isogenic pair, the APOE3ch cell line demonstrated a significant reduction in both Aβ40 and Aβ42 levels compared to the APOE3 cell line (Figures 3a, b). Despite these changes in Aβ levels, the Aβ42/Aβ40 ratio remained consistent across both the PS1*^E280A^* and APP*^dp^* isogenic pairs, suggesting that the APOE3ch modulates overall Aβ production without altering the relative balance of pathogenic Aβ species (Figure 3c).

**FIGURE 3:**
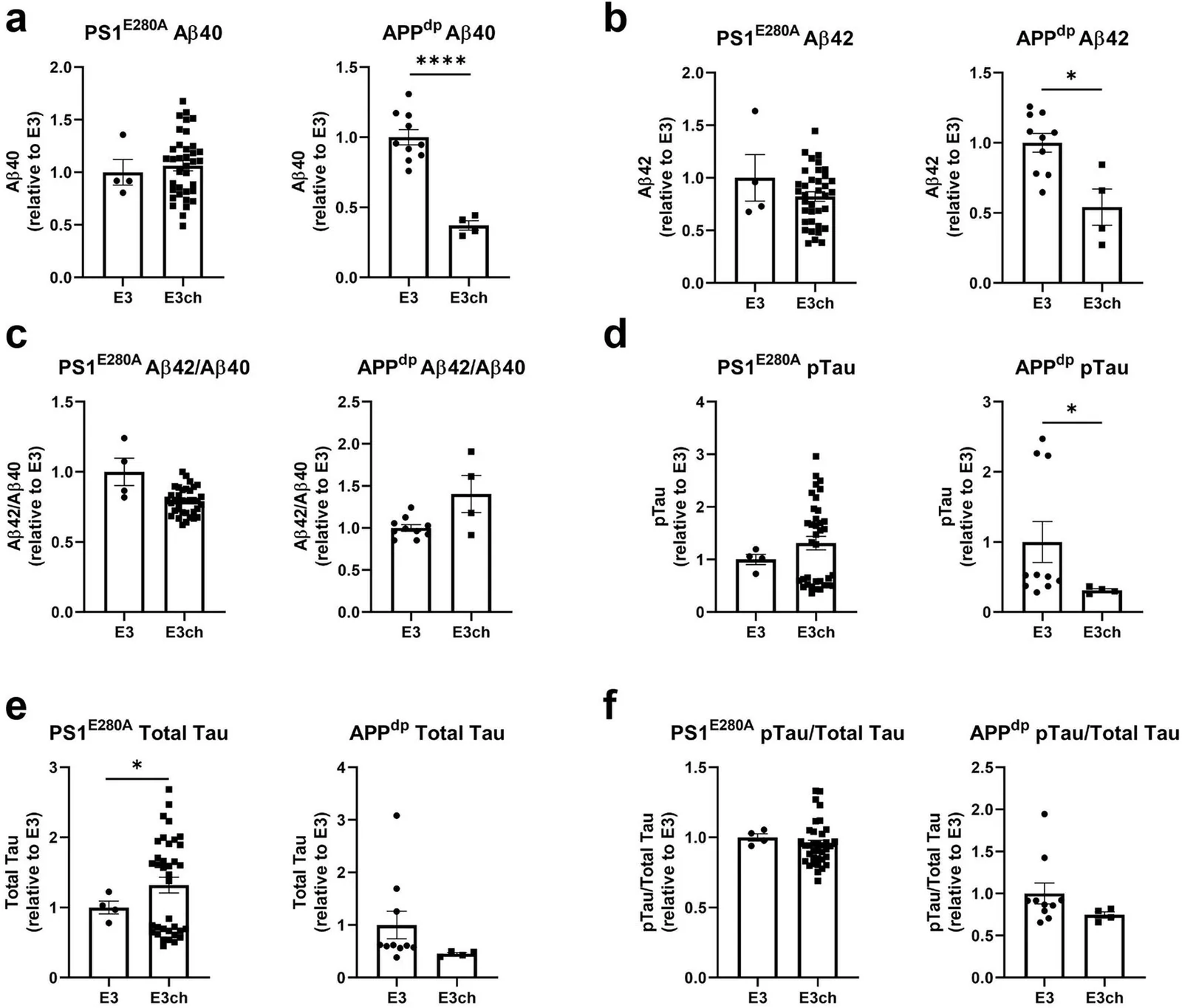
APOE3ch differentially modulates Aβ production and tau pathology across familial Alzheimer’s disease genetic backgrounds. **(a)** Quantification of secreted Aβ40 levels measured by ELISA from conditioned media of differentiated neural cultures derived from PSEN1 E280A (PS1^E280A^) and APP duplication (APP^dp^) APOE3 and APOE3ch isogenic clones. **(b)** Quantification of secreted Aβ42 levels measured by ELISA from conditioned media of differentiated neural cultures derived from PS1^E280A^ and APP^dp^ APOE3 and APOE3ch isogenic clones. **(c)** Ratio of Aβ42 to Aβ40 across all APOE3 and APOE3ch clones.**(d)** Quantification of phosphorylated tau (pTau; Thr231) levels in neural culture lysates measured by ELISA across all APOE3 and APOE3ch clones. **(e)** Quantification of total tau levels in neural culture lysates measured by ELISA across all APOE3 and APOE3ch clones.**(f)** Ratio of phosphorylated tau to total tau across all APOE3 and APOE3ch clones showing no significant genotype-dependent differences. All quantitative measurements are normalized to APOE3 levels within the respective parental cell line background. For ELISA data, replicates included PS1^E280A^ APOE3 (*n* = 4; 1 independent wells from differentiation #1, 1 independent well from differentiation #2, 2 independent wells from differentiation #3), PS1^E280A^ APOE3ch (*n* = 36; Clone 1: 6 independent wells from differentiation #1, 6 independent wells from differentiation #2, 8 independent wells from differentiation #3; Clone 2: 6 independent wells from differentiation #1, 1 independent well from differentiation #2, 2 independent wells from differentiation #3; Clone 3: 3 independent wells from differentiation #1, 2 independent wells from differentiation #2, 2 independent wells from differentiation #3), APP^dp^ APOE3 (*n* = 10; 1 independent well from differentiation #1, 6 independent wells from differentiation #2, 3 independent wells from differentiation #3), and APP^dp^ APOE3ch (*n* = 4; 2 independent wells from differentiation #1, 2 independent wells from differentiation #2). Statistical significance is indicated as follows: *p < 0.05, **p < 0.01, ***p < 0.001, ****p < 0.0001.

### 3.4 APOE3ch shows FAD mutation-specific effects on tau pathology in hiPSC-derived cultures

It has been shown that APOE4 promotes increased Aβ accumulation, which subsequently leads to tau pathology (Huang and Mucke, 2012). Indeed, this has been confirmed in hiPSC-based systems where isogenic neural cultures demonstrated that converting APOE3 and APOE4 resulted in elevated levels of both Aβ and phosphorylated tau (Zhao et al., 2020). On the other hand, work with isogenic hiPSC cultures demonstrates that converting APOE3 to APOE2 greatly reduced the production of Aβ (Brookhouser et al., 2021). The original case report described an individual with two copies of the APOE3ch mutation who exhibited limited tau pathology despite significant brain amyloid levels (Arboleda-Velasquez et al., 2019). Similarly, Wang et al. (2018) demonstrated in isogenic cultures that APOE4, compared to APOE3, increased tau phosphorylation levels without corresponding increases in Aβ production. These findings suggest that APOEch isoforms may modulate tau pathology through both amyloid-dependent and amyloid-independent mechanisms. To investigate this further, we assessed tau phosphorylation at Thr231, a key phosphoepitope, alongside total tau levels in cell lysates. Thr231 is a well-established Alzheimer’s disease-associated phosphoepitope that has been implicated in pathological tau assembly and has been used to evaluate tau-related phenotypes in human iPSC-derived neuronal models (Ashton et al., 2021; Israel et al., 2012; Potjewyd et al., 2022; Wang et al., 2007; Ye et al., 2021). In the PS1*^E280A^* APOE3ch clones, total tau levels significantly increased, while phosphorylated tau levels remained unchanged (Figure 3d). In contrast, the APP*^dp^* APOE3ch clones exhibited a significant reduction in phosphorylated tau levels compared to APOE3, with no changes in total tau levels (Figure 3e). The phosphorylated tau/total tau ratio showed no differences across either FAD mutation (Figure 3f). Collectively, these findings indicate that APOE3ch may influence Aβ and tau phenotypes in a FAD mutation-specific manner.

To investigate potential mechanisms underlying these mutation-specific tau phenotypes, we further analyzed gene expression using a curated set of genes associated with tau phosphorylation. This gene set was generated from an unbiased screen of human kinases capable of phosphorylating AD-associated tau epitopes, including Thr231 (Cavallini et al., 2013), together with genes annotated in the MSigDB Gene Ontology Molecular Function “tau protein kinase activity” gene set. Analysis of these tau-associated genes revealed distinct APOE3ch-dependent transcriptional changes across the two ADAD backgrounds (Supplementary Figure 7). In PS1*^E280A^* cultures, APOE3ch significantly altered expression of *CSNK1D*, *CDK17*, *MARK3*, and *DDR2*, whereas *CDK2* and *EIF2AK2* were significantly altered in APP*^dp^* cultures. Several of these genes have established roles in tau regulation. CSNK1D/CK1δ can directly phosphorylate tau and reduce its association with microtubules (Li et al., 2004), MARK-family kinases regulate tau–microtubule interactions and have been associated with early tau phosphorylation in AD (Lund et al., 2014), and EIF2AK2 has been identified as a regulator of phosphorylated and total tau levels (Azorsa et al., 2010). Notably, CDK2 and EIF2AK2 were both reduced in APP*^dp^* APOE3ch cultures, paralleling the reduction in pTau231 observed in this genetic background. CDK2 has also been identified a regulator of AD-associated tau phosphorylation, including phosphorylation involving Thr231 (Cavallini et al., 2013) In contrast, PS1*^E280A^* cultures exhibited a broader and more heterogeneous pattern of changes across several tau-associated kinases, consistent with the absence of a corresponding reduction in pTau231. Together, these findings identify distinct tau-regulatory transcriptional signatures in PS1*^E280A^* and APP*^dp^* cultures that may contribute to the mutation-dependent effects of APOE3ch on tau-related phenotypes.

### 3.5 APOE3ch modulates APP processing across FAD mutations

After synthesis, APP travels through the secretory pathway to the plasma membrane where is can undergoes non-amyloidogenic processing via α-secretase, releasing soluble APPα (sAPPα; Zhang et al., 2011). The resulting α-C-terminal fragment (α-CTF) is further cleaved by γ-secretase to produce the p3 peptide and the APP intracellular domain. Alternatively, APP not processed in this pathway is internalized via clathrin-mediated endocytosis, entering early endosomes (Marzolo and Bu, 2009). In the acidic environment of late endosomes, β-secretase cleavage produces soluble APPβ (sAPPβ) and the β-C-terminal fragment (β-CTF), which is subsequently cleaved by γ-secretase to generate Aβ. These Aβ peptides are then released into the extracellular space through exocytosis. Research on the effect of APOE isoforms on APP levels has been mixed with some studies showing that APOE elevates APP transcription and production in the order of E4 > E3 > E2 (Huang et al., 2017, 2019) whereas other studies have demonstrated no isoform-dependent effects of total APP levels (Wang et al., 2018). To determine the effect of the APOEch variant on APP levels, we measured total APP levels in our neural cultures across both FAD-related mutations. This analysis revealed no significant differences in total APP levels between the APOE3 and APOE3ch cultures (Figure 4a).

**FIGURE 4:**
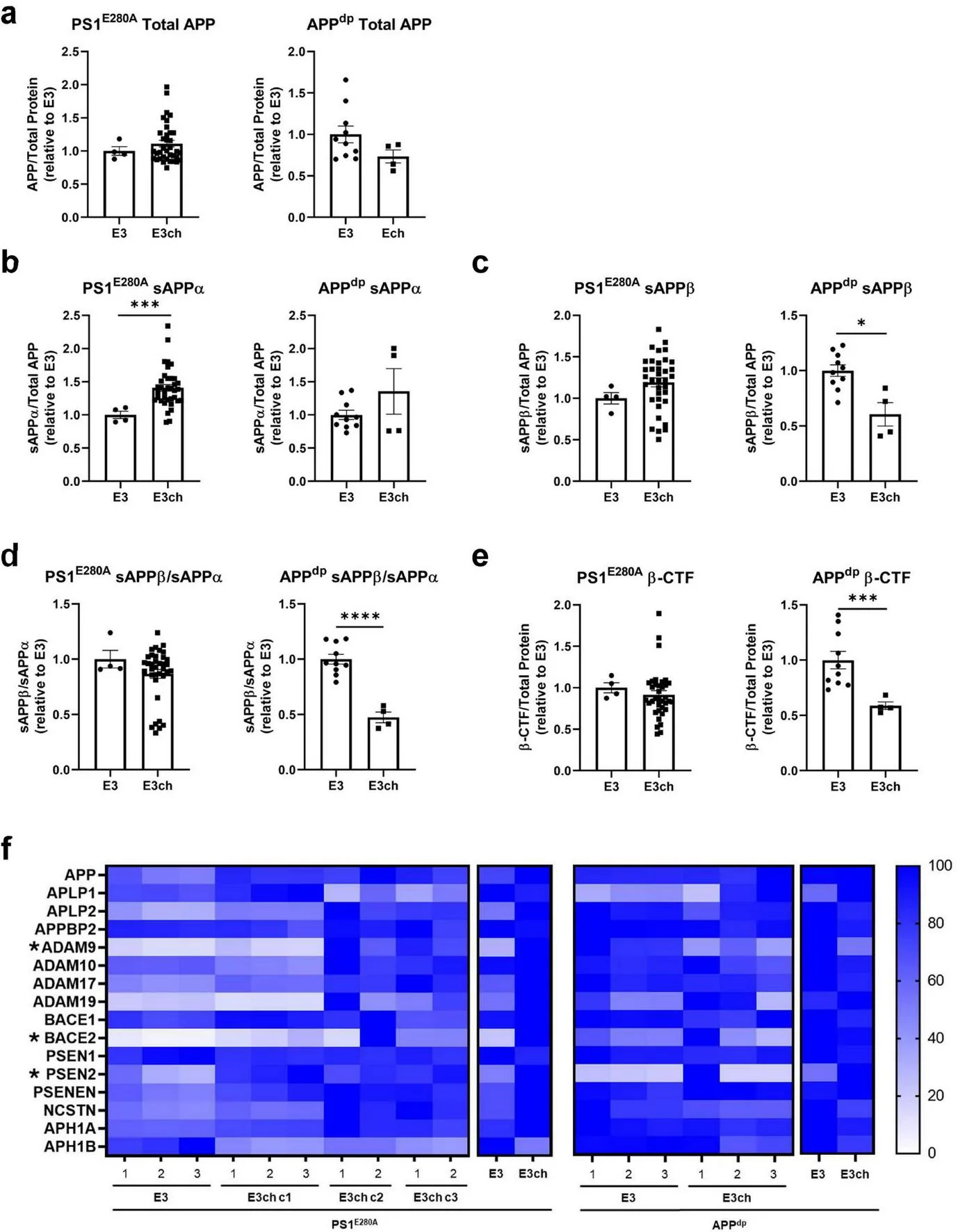
APOE3ch modulates APP processing in a familial Alzheimer’s disease mutation–dependent. manner. **(a)** Quantification of total APP protein levels in neural culture lysates from PSEN1 E280A (PS1^E280A^) and APP duplication (APPdp) APOE3 and APOE3ch isogenic clones measured by ELISA. **(b)** Quantification of soluble APPα (sAPPα) levels measured by ELISA from conditioned media of differentiated neural cultures. **(c)** Quantification of soluble APPβ (sAPPβ) levels measured by ELISA from conditioned media of differentiated neural cultures. **(d)** Ratio of sAPPβ to sAPPα across PS1^E280A^ and APP^dp^ APOE3 and APOE3ch isogenic clones. **(e)** Quantification of β-C-terminal fragment (β-CTF) levels measured in neural culture lysates by ELISA. Statistical significance is indicated as follows: *p < 0.05, **p < 0.01, ***p < 0.001, ****p < 0.0001. **(f)** Gene expression analysis of APP processing–related transcripts, including APP, ADAM family α-secretases, BACE1/BACE2 β-secretases, and γ-secretase complex components across isogenic neural cultures. Significant genes in PSEN1 E280A neural cultures are indicated by an asterisk (*). All quantitative measurements were normalized to APOE3 levels within the corresponding parental cell line background. For RNA-seq data, each column represents an independent well from independent differentiations. For ELISA data, replicates included PS1^E280A^ APOE3 (*n* = 4; 1 independent wells from differentiation #1, 1 independent wells from differentiation #2, 2 independent wells from differentiation #3), PS1^E280A^ APOE3ch (*n* = 36; Clone 1: 6 independent wells from differentiation #1, 6 independent wells from differentiation #2, 8 independent wells from differentiation #3; Clone 2: 6 independent wells from differentiation #1, 1 independent well from differentiation #2, 2 independent wells from differentiation #3; Clone 3: 3 independent wells from differentiation #1, 2 independent wells from differentiation #2, 2 independent wells from differentiation #3), APP^dp^ APOE3 (*n* = 10; 1 independent well from differentiation #1, 6 independent wells from differentiation #2, 3 independent wells from differentiation #3), and APP^dp^ APOE3ch (*n* = 4; 2 independent wells from differentiation #1, 2 independent wells from differentiation #2).

Previous studies have shown that APOE modulates differences in amyloidogenic versus non-amyloidogenic processing of APP (Small et al., 2017). To determine if APOE3ch influences APP processing, we measured the levels of soluble APP (sAPP) fragments secreted into culture medium generated by APP cleavage due to the amyloidogenic (sAPPβ) or non-amyloidogenic (sAPPα) pathway. In the PS1*^E280A^* isogenic line, APOE3ch cultures showed a significant increase in sAPPα levels compared to APOE3 neurons, with no change in sAPPβ levels (Figures 4b, c). Conversely, in the APP*^dp^* cell lines, no significant difference in sAPPα levels was observed, but sAPPβ levels were significantly reduced in the APOE3ch line relative to APOE3 (Figures 4b, c). Furthermore, the sAPPβ/sAPPα ratio was significantly decreased in the APPdp APOE3ch line, with no notable change in this ratio in the PS1*^E280A^* line (Figure 4d). To further investigate amyloidogenic APP processing, we analyzed levels of β-CTF in cell lysates. While no differences were observed between APOE3 and APOE3ch in the PS1*^E280A^* isogenic lines, the APP*^dp^* APOE3ch line exhibited a significant reduction in β-CTF levels compared to APOE3 (Figure 4e). Together, this suggests that the reduced levels of total Aβ observed in the APP*^dp^* APOE3ch cultures is due to a reduction of amyloidogenic processing of APP.

Next, we measured the effect of the APOEch mutation on APP processing at the transcription level (Figure 4f). In line with our previous findings, we observed no significant effect of APOEch on APP transcription across the FAD mutations. Since α-secretases are part of the ADAM family and β-secretases are encoded by the *BACE1* and *BACE2* genes, we analyzed the expression levels of ADAMs and BACEs in the isogenic cultures. Gene expression analysis revealed a notable increase in *ADAM9* expression in PS1E280A APOE3ch cultures, along with elevated *BACE2* levels. γ-secretase is composed of PSEN, NCSTN, PEN2, and APH1 proteins (Kimberly et al., 2003). With two *PSEN* genes (*PSEN1, PSEN2*), two *APH1* genes (*APH1A*, *APH1B*), and multiple *APH1* isoforms, γ-secretase complexes can exist in diverse forms, thereby influencing APP processing. Although expression of most of the γ-secretase components was similar between the APOE3 and APOE3ch cultures, there was a significant difference in expression of *PSEN2* in the PS1*^E280^* isogenic cultures. Altogether, these findings suggest that the APOE3ch variant modulates APP processing at both transcriptional and post-translational levels in a FAD mutation-dependent manner, potentially contributing to its protective effects in AD.

### 3.6 APOEch alters transcriptional patterns in hiPSC-derived neural cultures

Recent studies demonstrating that APOE exerts isoform-specific effects on various downstream signaling pathways suggests that various APOE isoforms might more broadly influence the transcriptional profile of target cell populations (Xu et al., 2006, 2007). To that end, we analyzed the broader gene expression profiles of iPSC-derived neurons to identify molecular pathways influenced by the APOE3ch variant. To assess transcriptional differences, we compared differentially expressed genes (DEGs) in two FAD isogenic cell lines, normalizing DEGs to the APOE3 cell line control (Supplementary Data 1). In the PS1*^E280A^* isogenic line, 2,296 DEGs were identified, including 1,628 upregulated and 668 downregulated genes. In contrast, the APP*^dp^* cell lines showed 780 DEGs, with 522 upregulated and 258 downregulated genes (Figure 5a). We also examined the overlap of DEGs between the two FAD mutations, identifying 292 shared genes that were differentially expressed in the presence of the APOE3ch variant. Additionally, 2,004 unique DEGs were specific to the PS1*^E280A^* isogenic line, while 488 unique DEGs were found in the APP*^dp^* isogenic line, highlighting mutation-specific transcriptional profiles (Figure 5b).

**FIGURE 5:**
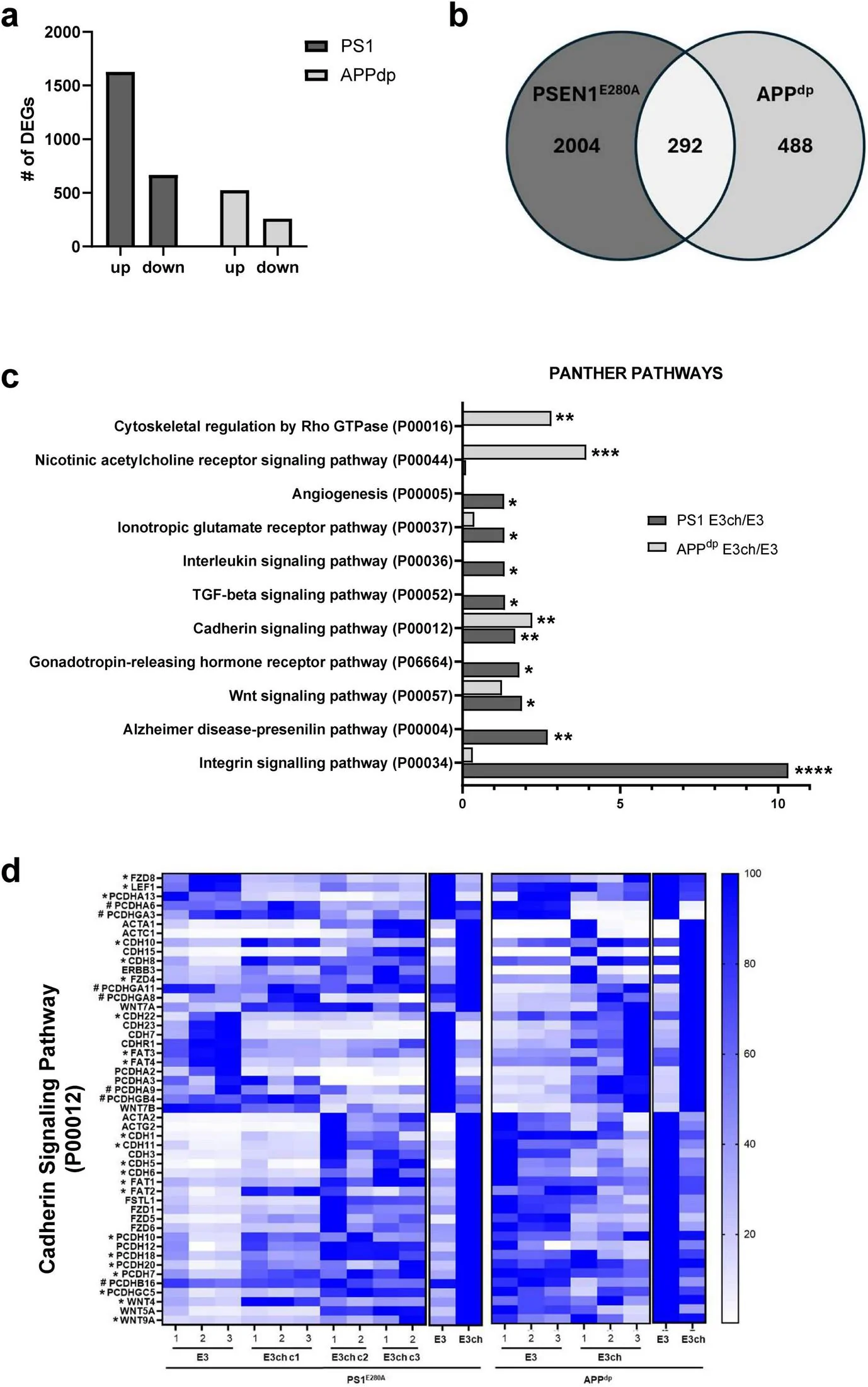
APOE3ch induces mutation-specific transcriptional changes and pathway alterations in hiPSC-derived neural cultures. **(a)** Quantification of the number of upregulated and downregulated differentially expressed genes (DEGs) in APOE3ch neural cultures relative to APOE3 controls in PSEN1 E280A (PS1^E280A^) and APP duplication (APP^dp^) isogenic lines (| log2 fold change | > 1 and adjusted *p*-value < 0.05). **(b)** Venn diagram illustrating the overlap of DEGs shared between PSEN1 E280A and APPdp APOE3ch neural cultures. **(c)** Pathway enrichment analysis showing differentially expressed signaling pathways across PSEN1 E280A and APPdp neural cultures. Statistical significance is indicated as follows: *p < 0.05, **p < 0.01, ***p < 0.001, ****p < 0.0001. **(d)** Heatmap of cadherin signaling pathway gene expression across PSEN1 E280A and APPdp APOE3 and APOE3ch neural cultures. Significance in PSEN1 E280A cultures is denoted by an asterisk (*), and significance in APPdp cultures is denoted by a number sign (#). For RNA-seq data, each column represents an independent well from independent differentiations.

To further analyze DEGs in the context of both FAD mutations, we mapped them to relevant pathways using PANTHER (Mi et al., 2013). This analysis identified several statistically significant pathways uniquely influenced by the APOE3ch variant in specific FAD mutations (Figure 5c). Notably, the APOE3ch variant significantly impacted the Cadherin signaling pathway across both mutations. Further examination of Cadherin pathway-specific genes revealed that while some were consistently affected in the same direction, others exhibited opposing effects depending on the mutation (Figure 5d). This suggests that APOE3ch’s influence on Cadherin signaling is mutation-dependent, potentially altering interactions with key pathway components and leading to distinct downstream effects.

Beyond the Cadherin pathway, PANTHER analysis revealed mutation-specific effects on additional signaling pathways. In the APP^dp^ mutation, APOE3ch significantly influenced cytoskeletal regulation by Rho GTPase and nicotinic acetylcholine receptor signaling. In contrast, in the PS1^E280A^ mutation, APOE3ch significantly affected angiogenesis, inotropic glutamate receptor signaling, interleukin signaling, TGF-beta signaling, gonadotropin-releasing hormone receptor signaling, Wnt signaling, Alzheimer disease-presenilin signaling, and integrin signaling. Overall, these findings highlight the complexity of APOE3ch’s role in modulating disease-related pathways and suggest that its effects are highly context-dependent.

To further identify potential regulatory mechanisms underlying these transcriptional changes, we performed Ingenuity Pathway Analysis (IPA) upstream regulator analysis (Supplementary Data 2). This analysis revealed, 1,406 significant upstream regulators were identified in PS1^E280A^ cultures compared with only 86 in APP^dp^ cultures, consistent with the substantially greater number of DEGs and broader spectrum of altered pathways observed in the PS1^E280A^ background. The PS1^E280A^ regulatory profile included strong predicted activation of several inflammatory and stress-associated regulators, including TNF, IFNG, IL1B, NFκB-associated factors, and TGFB1, consistent with the effects on interleukin and TGF-β signaling identified by PANTHER. Several regulators related to APP/Aβ biology were also identified, including predicted activation of HIF1A and APP. HIF1A has previously been linked to regulation of BACE1 expression and γ-secretase activity under hypoxic conditions, thereby connecting this upstream-regulator profile with pathways involved in amyloidogenic APP processing (Alexander et al., 2022; Chen et al., 2013; Guglielmotto et al., 2009a). In contrast, the substantially smaller APP^dp^ upstream-regulator set included predicted changes in transcriptional and epigenetic regulators such as SIX1, KDM5A, DNMT3A, and DNMT3B (Chen et al., 2025; Toth et al., 2025; Wu et al., 2015) consistent with the more restricted transcriptional and pathway-level response observed in this genetic background.

To more specifically examine upstream regulatory changes relevant to AD, we next compared the significant IPA regulators with the AD-associated transcriptional regulators and signaling molecules compiled by Chen et al. (2013). This refined analysis identified 33 significant AD-associated upstream regulators in PS1^E280A^ cultures compared with only three in APP^dp^ cultures. Notably, IL1B and PPARGC1A were the only AD-associated regulators significantly altered in both genetic backgrounds, but their predicted activities were opposite as IL1B was activated and PPARGC1A inhibited in PS1^E280A^ cultures, whereas IL1B was inhibited and PPARGC1A activated in APP^dp^ cultures. IL1B is a central mediator of inflammatory signaling implicated in AD-related neuroinflammation (Lopez-Rodriguez et al., 2021), whereas PPARGC1A/PGC-1α regulates mitochondrial biogenesis, oxidative metabolism, and cellular responses to oxidative stress (Sweeney and Song, 2016) Additional PS1^E280A^-associated changes included predicted activation of HIF1A, APP, NFκB-related regulators, CREB1, and NGF together with predicted inhibition of APOE and BACE1. In contrast, the three AD-associated regulators identified in APP^dp^ cultures consisted of predicted activation of PPARGC1A and BDNF and inhibition of IL1B. BDNF is an established regulator of neuronal survival and synaptic plasticity (Gao et al., 2022; Lu et al., 2014). These contrasting upstream-regulator profiles parallel the phenotypic and transcriptional differences observed earlier in the study. Specifically, APOE3ch produced a broader transcriptional response but comparatively modest effects on extracellular Aβ and APP processing in PS1^E280A^ cultures, whereas APP^dp^ cultures exhibited a more restricted transcriptional response accompanied by reductions in amyloidogenic APP processing, extracellular Aβ, and pTau231. Together, these findings demonstrate that APOE3ch produces both quantitatively broader and qualitatively distinct regulatory responses depending on the underlying ADAD mutation.

## 4 Discussion

In this study, we used isogenic hiPSC-derived neural models to investigate how the rare APOE3 Christchurch (APOE3ch) variant modulates Alzheimer’s disease (AD)–related phenotypes across two distinct familial AD (FAD) mutations–PSEN1 E280A (PS1^E280A^) and APP duplication (APP^dp^). This work builds on the original clinical report describing a homozygous APOE3ch carrier who remained cognitively intact for decades beyond the expected age of AD onset, despite harboring the highly penetrant PSEN1 E280A mutation (Arboleda-Velasquez et al., 2019). Neuropathological examination later confirmed extensive amyloid deposition but remarkably limited tau pathology and neurodegeneration (Sepulveda-Falla et al., 2022), suggesting that APOE3ch confers protection downstream of amyloid accumulation. Although subsequent studies showed that APOE3ch reduces binding to heparan sulfate proteoglycans (HSPGs) and attenuates tau uptake, Aβ aggregation, and neurodegeneration (Arboleda-Velasquez et al., 2019; Marino et al., 2024; Nelson et al., 2023; Sun et al., 2024; Perez-Corredor et al., 2025; Murakami et al., 2024; Tran et al., 2025), the extent to which APOE3ch functions across different genetic backgrounds and whether it alters APP processing or broader transcriptional programs has remained unclear. By examining APOE3ch across two genetically distinct ADAD mutations in hiPSC-derived neurons and astrocytes, our study fills this critical gap and provides new mechanistic insights into how APOE3ch influences AD-related pathways in a mutation-specific manner.

Across both PS1^E280A^ and APP^dp^ backgrounds, APOE3ch did not alter neuronal or astrocytic differentiation, the specification of neuronal subtypes, or expression of synaptic gene markers. Likewise, APOE3ch did not affect APOE secretion, consistent with prior work demonstrating that APOE stability and secretion are primarily isoform-dependent (E4 < E3 < E2) (Bien-Ly et al., 2012; Wang et al., 2018) and that the Christchurch R136S substitution likely influences receptor-binding and HSPG interactions rather than secretion pathways. This distinguishes APOE3ch from other APOE variants that can reduce APOE protein levels or lipidation efficiency in some contexts (Gao et al., 2018). Together, these findings indicate that APOE3ch-mediated protection arises not from altered APOE abundance but from downstream signaling changes or disrupted protein–protein interactions such as reduced HSPG engagement.

Previous hiPSC-based APOE3ch studies have primarily examined tau pathology without assessing Aβ production or dissecting APP processing pathways (Arboleda-Velasquez et al., 2019; Marino et al., 2024; Nelson et al., 2023; Sun et al., 2024; Perez-Corredor et al., 2025; Murakami et al., 2024; Tran et al., 2025; Perez-Corredor et al., 2024). To that end, a major advance from our work is the discovery that APOE3ch reduces total Aβ40 and Aβ42 production exclusively in the APP^dp^ background, with no corresponding effect observed in PSEN1 E280A cultures. Consistent with this, we found that APOE3ch shifts APP processing away from amyloidogenic pathways in APP^dp^ cultures–specifically decreasing sAPPβ, β-CTF, and sAPPβ/sAPPα ratios, a profile indicative of reduced β-secretase–dependent processing. In contrast, APOE3ch increased sAPPα levels in PS1^E280A^ cultures without altering amyloidogenic fragments. Importantly, extracellular Aβ levels reflect not only APP processing and Aβ generation, but also trafficking, cellular uptake, degradation, and clearance. Thus, our measurements of secreted Aβ cannot distinguish whether the observed reductions arise exclusively from altered production or also involve changes in Aβ turnover. This distinction is important because APOE isoforms regulate soluble Aβ clearance and extracellular metabolism in an isoform-dependent manner (Castellano et al., 2011; DeMattos et al., 2004). Moreover, the ELISA measurements used here quantify total extracellular Aβ40 and Aβ42 and do not distinguish among monomeric, oligomeric, or higher-order aggregated Aβ species. This is an important limitation because soluble Aβ oligomers have been strongly implicated in synaptic dysfunction and neurotoxicity in AD, and total Aβ abundance is therefore not necessarily proportional to the abundance or biological activity of pathogenic Aβ assemblies (Ghosh et al., 2023; Selkoe and Hardy, 2016).

Mechanistically, APP duplication expands total APP substrate availability, rendering APP processing more sensitive to perturbations in lipid metabolism, receptor trafficking, and endocytic pathways (O’Brien and Wong, 2011; Zhang et al., 2011). This increased dependence on trafficking and endocytic regulation is important because the same receptor systems that govern APP internalization also participate in downstream Aβ handling. LRP1 and related APOE receptors can influence APP internalization and amyloidogenic processing while also mediating Aβ uptake and clearance, thereby linking APP trafficking with Aβ turnover (Kanekiyo and Bu, 2014; Kanekiyo et al., 2013; Van Gool et al., 2019). HSPGs further contribute to this network by interacting with LRP1 and other cell-surface receptors to regulate APOE and Aβ binding, internalization, and clearance. As a result, APOE-dependent effects on APP processing are difficult to disentangle from effects on extracellular Aβ trafficking and clearance (Kanekiyo and Bu, 2014; Ozsan McMillan et al., 2023).

Together, these established links among APP processing, APOE receptor biology, HSPG interactions, and Aβ turnover provide a mechanistic framework for interpreting the genotype-specific effects observed in our cultures. Within this framework, our findings support a model in which the APOE3ch-associated reduction in HSPG binding (Arboleda-Velasquez et al., 2019) alters receptor-dependent trafficking of APOE, APP, and/or Aβ, with these effects becoming more evident in an APP-overexpressing system. This interpretation is also consistent with the location of the APOE3ch R136S substitution within the HSPG/receptor-binding region of APOE and with evidence that APOE-HSPG interactions regulate APOE uptake and AD-associated protein trafficking (Fu et al., 2016; Holmes et al., 2013; Raulin et al., 2022; Reinhard et al., 2013; Zhao et al., 2018). Accordingly, the reduction in sAPPβ, β-CTF, and extracellular Aβ observed in APP^dp^ cultures may reflect altered APP trafficking and processing, altered downstream Aβ turnover, or a combination of these mechanisms rather than a simple reduction in Aβ production. Likewise, although reduced total extracellular Aβ40 and Aβ42 may be consistent with an amyloid-modifying effect of APOE3ch, these data do not establish that APOE3ch reduces the abundance or toxicity of oligomeric Aβ species. Direct assessment of oligomeric Aβ, together with functional measures of neuronal or synaptic toxicity, will be needed to determine whether the changes observed here translate into reduced Aβ pathogenicity.

This distinction may also help reconcile our findings with the original APOE3ch case report. Although APOE3ch was shown to reduce Aβ42 aggregation *in vitro*, the APOE3ch homozygous PS1^E280A^ carrier nevertheless exhibited substantial cerebral amyloid deposition (Arboleda-Velasquez et al., 2019). These observations suggest that APOE3ch may influence specific aspects of Aβ aggregation, trafficking, or turnover without necessarily preventing overall amyloid accumulation. Accordingly, measurements of total extracellular Aβ alone may not capture APOE3ch-dependent changes in the composition, aggregation state, or biological activity of the Aβ pool.

The absence of this effect in PS1^E280A^ cultures suggests that when γ-secretase activity is directly altered by PSEN1 mutations (Kelleher and Shen, 2017), upstream modulation by APOE3ch is insufficient to reduce net extracellular Aβ levels. Nevertheless, we observed a modest increase in non-amyloidogenic processing in PS1^E280A^ APOE3ch cultures, reflected by elevated sAPPα without changes in sAPPβ or β-CTF. This shift aligns with reports that APOE variants can influence APP trafficking and α-secretase activity (Lee et al., 2017; Raulin et al., 2022). Supporting this, we detected increased ADAM9 (α-secretase) levels in PS1^E280A^ APOE3ch cultures. Taken together, these results expand the mechanistic framework of APOE3ch-mediated protection, demonstrating that its effects may include genotype-specific modulation of APP processing and Aβ generation. Future studies directly measuring APP internalization, Aβ uptake and degradation, and receptor/HSPG-dependent trafficking will be necessary to determine the relative contributions of altered APP processing versus Aβ clearance and turnover to the APOE3ch phenotype. In parallel, biochemical quantification of oligomeric Aβ species and functional assays of their neurotoxic and synaptotoxic effects will be required to determine whether APOE3ch alters the pathogenic composition of the Aβ pool in addition to its effects on total extracellular Aβ levels.

In this study, tau-related phenotypes differed depending on the underlying ADAD mutation. In PS1^E280A^ neural cultures, the APOE3ch variant did not alter phosphorylated tau at Thr231, although it modestly increased total tau levels, indicating that APOE3ch does not substantially alter pTau231 levels in this genetic context. In contrast, APP^dp^ cultures carrying the APOE3ch variant exhibited a significant reduction in pTau231 without changes in total tau, suggesting a reduction in this specific tau phosphorylation marker rather than a generalized effect on tau pathology. Across both mutation backgrounds, the ratio of phosphorylated to total tau remained unchanged, suggesting that APOE3ch influences the specific tau-related phenotypes measured here in a mutation-dependent manner. In relation to tau, these findings are partially aligned with the original APOE3ch case report (Arboleda-Velasquez et al., 2019) and subsequent hiPSC-based studies (Murakami et al., 2024; Nelson et al., 2023; Perez-Corredor et al., 2024). The original report showed that the APOE3ch homozygous PSEN1^E280A^ carrier displayed limited tau pathology despite extensive amyloid deposition (Arboleda-Velasquez et al., 2019). In contrast, our PS1^E280A^ hiPSC-derived cultures did not exhibit reductions in p-tau (Thr231). Instead, only total tau increased modestly. By comparison, in the APP^dp^ background APOE3ch reduced pTau231 levels without affecting total tau, consistent with a genotype-specific effect on this tau phosphorylation site. This is consistent with other studies showing that APOE3ch’s effects on tau-related phenotypes become most apparent in the context of elevated Aβ levels (Chen et al., 2024). The study by Perez-Corredor et al. (2024) reported reductions in tau phosphorylation in PS1^E280A^ organoids, whereas our 2D neuron–astrocyte PS1^E280A^ cultures did not show a reduction in pTau231. Because the two studies assessed different tau phosphorylation epitopes, these findings are not directly comparable. Differences in the specific phospho-tau sites examined, as well as 3D versus 2D culture architecture and assay sensitivity, may contribute to the distinct tau-related phenotypes observed across the two studies. Similarly, Nelson et al. (2023) demonstrated that introducing the R136S mutation into APOE4 backgrounds robustly rescues APOE4-driven tau pathology in mouse models and human iPSC-derived neurons, suggesting a broad tau-protective capacity of APOE3ch/R136S in a late-onset AD risk context. In contrast, our findings indicate that APOE3ch-associated changes in pTau231 and total tau are conditional on the underlying genetic background, with reduced pTau231 observed in APP^dp^ cultures but minimal effects on pTau231 in PS1^E280A^ cultures. Other hiPSC studies (Murakami et al., 2024; Nelson et al., 2023; Sun et al., 2024) have emphasized that APOE3ch’s effects may relate less to tau phosphorylation *per se* and more to its role in regulating tau accumulation, uptake, and propagation, often through APOE–HSPG interactions.

Because the present study evaluated only pTau231, additional disease-relevant phospho-tau epitopes should be examined in future work to determine whether APOE3ch differentially affects distinct stages or conformational states of tau pathology. In particular, pTauS396 would be informative because phosphorylation at Ser396 is enriched in pathological tau isolated from AD brain, contributes to impaired microtubule binding, and is associated with paired helical filament tau (Basheer et al., 2023; Bramblett et al., 1993). Similarly, AT8, which recognizes tau phosphorylated at Ser202/Thr205, is widely used to identify AD-associated pathological tau and neurofibrillary pathology (Goedert et al., 1995; Malia et al., 2016). Future examination of there other AD-relevant phosphorylation sites may therefore provide a more comprehensive understanding of how APOE3ch influences different tau-related phenotypes across genetic backgrounds. Nonetheless, taken together, previous findings and the present work demonstrate that while APOE3ch can mitigate pathogenic tau processes, its influence is shaped by the upstream ADAD mutation, the specific cellular environment, and the balance between amyloidogenic and non-amyloidogenic signaling pathways.

The tau-focused transcriptomic analysis provides additional insight into potential mechanisms underlying these mutation-dependent phenotypes. APOE3ch altered distinct sets of tau-associated kinases in the two ADAD backgrounds, with significant changes in *CSNK1D*, *CDK17*, *MARK3*, and *DDR2* in PS1^E280A^ cultures but *CDK2* and *EIF2AK2* in APP^dp^ cultures. This finding is consistent with the broad kinase network known to regulate tau phosphorylation, including multiple kinases capable of modifying AD-associated epitopes such as Thr231 (Cavallini et al., 2013). Of particular interest, CDK2 and EIF2AK2 were reduced in APP^dp^ APOE3ch cultures, the same background in which we observed decreased pTau231. Because CDK2 has been identified as a kinase capable of regulating AD-associated tau phosphorylation, including Thr231-related phosphorylation, reduced CDK2-associated signaling represents one potential mechanism contributing to the lower pTau231 phenotype in APP^dp^ cultures. In contrast, PS1^E280A^ cultures exhibited a broader and mixed pattern of tau-regulatory kinase changes, which may help explain why substantial transcriptional remodeling in this background did not translate into a net reduction in pTau231. These observations further support a model in which APOE3ch modifies tau-regulatory networks in a manner that depends on the underlying ADAD mutation rather than uniformly suppressing tau phosphorylation. However, because transcript abundance does not directly establish kinase activity, phosphorylation state, or direct kinase–tau interactions, these genes should be considered candidate mediators that will require biochemical and functional validation.

Although the phenotypic effects of APOE3ch on Aβ and tau were clearly mutation dependent, transcriptional profiling revealed convergent pathway-level changes, particularly in Wnt and cadherin signaling. In both PS1E280A and APPdp isogenic sets, APOE3ch neural cultures exhibited distinct gene expression signatures relative to APOE3 controls, with upregulated expression of genes linked to Wnt/β-catenin and cadherin pathways. These pathways are increasingly recognized as critical regulators of synaptic integrity, neuronal survival, and AD pathogenesis. Aberrant Wnt signaling has been implicated in Aβ production, tau phosphorylation, and synapse loss in multiple models, and recent work in AD cerebral organoids has highlighted Wnt pathway dysregulation as a core feature of disease progression (Kostes and Brafman, 2023; Tapia-Rojas and Inestrosa, 2018). Recent cerebral organoid studies demonstrated that APOE3ch modulates β-catenin/Wnt and cadherin pathways in PS1*^E280A^* backgrounds, mirroring our findings in 2-D neural cultures (Perez-Corredor et al., 2024). Cadherin signaling has also been linked to synaptic adhesion stability and neurodegeneration (Ando et al., 2011; Arikkath and Reichardt, 2008; Laszlo and Lele, 2022; Mysore et al., 2008), and APOE isoforms differentially modulate components of cadherin–catenin complexes (Ahmad et al., 2020; Zhang et al., 2024). The consistent upregulation of Wnt and cadherin signaling across both FAD mutations suggests a potential shared mechanism underlying APOE3ch-mediated synaptic resilience.

At the same time, APOE3ch induced extensive yet highly mutation-specific transcriptional changes in hiPSC-derived neural cultures, revealing that its impact on cellular signaling networks depends strongly on the underlying ADAD mutation. PS1^E280A^ cultures exhibited more than twice as many differentially expressed genes as APP^dp^ cultures, together with a broader spectrum of perturbed pathways. This distinction was reinforced by IPA, which identified 1,406 significant upstream regulators in PS1^E280A^ compared with only 86 in APP^dp^ cultures and, after refinement using AD-associated regulators described by Chen et al. 33 AD-related regulators in PS1^E280A^ compared with only three in APP^dp^. The PS1^E280A^ profile included predicted activation of inflammatory and stress-associated regulators such as IL1B, NFκB-related factors, HIF1A, and APP, together with predicted inhibition of APOE, BACE1, and PPARGC1A. This broad and mixed response is consistent with the wider perturbation of interleukin, TGF-β, Wnt, glutamatergic, and presenilin signaling observed in our pathway analysis and with previous evidence that presenilins influence synaptic function and intracellular calcium homeostasis (Ho and Shen, 2011; Shen and Kelleher, 2007). Notably, predicted HIF1A activation may provide an additional link to APP/Aβ biology because HIF1A has been shown to increase BACE1 expression and γ-secretase activity under hypoxic conditions (Alexander et al., 2022; Guglielmotto et al., 2009b). However, the simultaneous predicted inhibition of BACE1 and the absence of reductions in sAPPβ, β-CTF, or extracellular Aβ indicate that these IPA predictions do not reflect a simple coordinated shift toward or away from amyloidogenic processing. Rather, APOE3ch may broadly reorganize signaling networks in PS1^E280A^ cells without overcoming the dominant consequences of altered presenilin/γ-secretase function.

In contrast, the APP^dp^ upstream-regulator profile was considerably more restricted and was characterized by predicted activation of PPARGC1A and BDNF together with inhibition of IL1B. PPARGC1A/PGC-1α regulates mitochondrial function and cellular responses to oxidative stress and has been implicated in AD-associated metabolic dysfunction (Sweeney and Song, 2016). BDNF is a major regulator of neuronal survival and synaptic plasticity and has been linked to synaptic dysfunction in AD (Gao et al., 2022; Lu et al., 2014), whereas IL1B is a major mediator of AD-associated neuroinflammatory signaling (Lopez-Rodriguez et al., 2021). Thus, the combination of predicted PPARGC1A and BDNF activation with IL1B inhibition raises the possibility that APOE3ch promotes a transcriptional state favoring metabolic and neurotrophic support while reducing inflammatory signaling in the APP^dp^ background. Importantly, this comparatively focused transcriptional response coincided with the strongest biochemical phenotype in our study, including reductions in sAPPβ, β-CTF, extracellular Aβ, and pTau231. Because APP and BACE1 were not identified as significant AD-associated upstream regulators in APP^dp^ cultures, these biochemical effects are unlikely to result solely from transcriptional suppression of APP or β-secretase pathways. Instead, they are consistent with the receptor trafficking, endocytic, and HSPG-dependent mechanisms proposed above, whereby reduced APOE3ch-HSPG binding may alter APP trafficking and/or downstream Aβ turnover. Together, these findings suggest that APOE3ch engages a broad signaling response in the PS1^E280A^ background but a more focused metabolic, neurotrophic, and inflammatory regulatory program in APP^dp^ cells, further supporting the conclusion that its protective mechanisms are strongly dependent on the underlying ADAD mutation.

In summary, our study shows that the rare APOE3 Christchurch variant acts as a context-dependent modifier of Alzheimer’s disease rather than a universal suppressor of pathology. Using isogenic hiPSC-derived neural models, we demonstrate that APOE3ch reduces amyloidogenic APP processing, Aβ production, and tau phosphorylation in APP duplication backgrounds, while primarily modulating non-amyloidogenic processing and transcriptional programs linked to synaptic signaling in PS1^E280A^ cultures. Despite these mutation-specific phenotypic effects, APOE3ch consistently converged on pathways associated with synaptic function and potential resilience, including Wnt and cadherin signaling. Indeed, given recent questions regarding the strength and interpretation of some of the human genetic evidence supporting APOE3ch-mediated protection, future independent mechanistic studies across multiple experimental models and research groups will be important for defining the robustness and context dependence of its protective effects (Piller and Smith, 2026). In the future, direct functional studies under disease-relevant stress conditions will be important to determine whether these pathway-level changes confer measurable synaptic resilience. Together, these findings provide a mechanistic framework for APOE3ch-mediated resilience and highlight the importance of genetic context in shaping the impact of protective variants, with implications for precision therapeutic strategies in Alzheimer’s disease.

## Data Availability

The raw data supporting the conclusions of this article will be made available by the authors, without undue reservation. The RNAseq datasets generated in this study have been deposited in the NCBI Gene Expression Omnibus (GEO) under accession number GSE338592: https://www.ncbi.nlm.nih.gov/geo/query/acc.cgi.
