# Supplementary material for: Isogenic human stem cell models reveal mutation-dependent protective mechanisms of APOE3 Christchurch
Source: Front Aging Neurosci. 2026 Oct 1;18:1856154. doi: 10.3389/fnagi.2026.1856154 (PMC13669193; doi:10.3389/fnagi.2026.1856154)
Supplement: Supplementary file 1 [file Data_Sheet_1.pdf]

SUPPLEMENTAL FIGURES

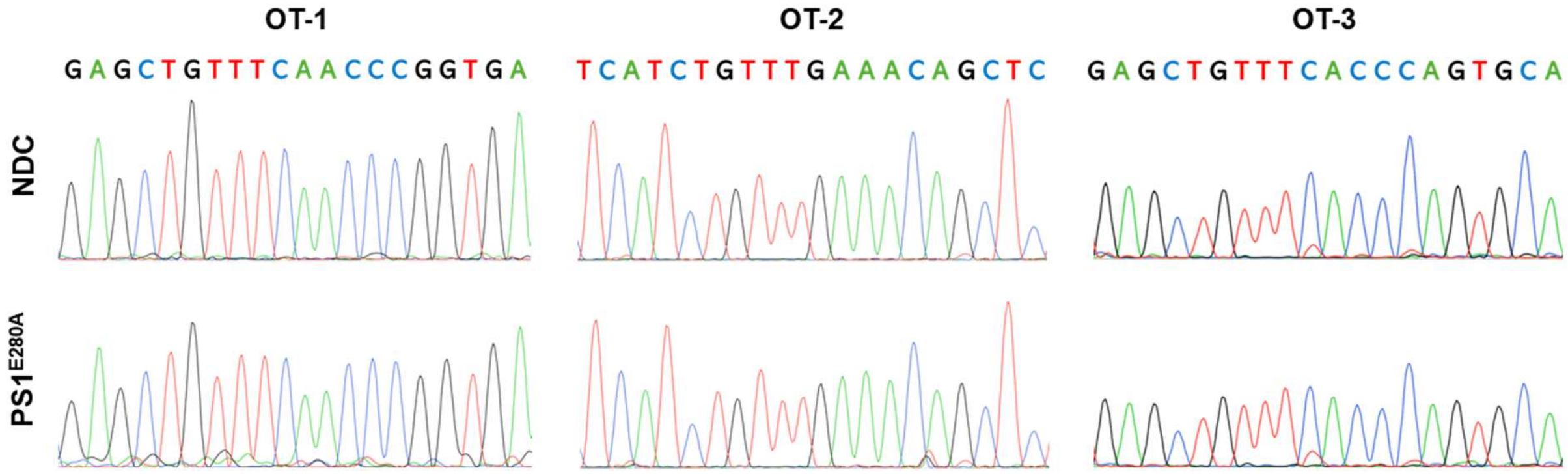

Supplemental Figure 1. Off-target analysis of PS1^{E280A} prime-edited hiPSC clones. Representative Sanger sequencing chromatograms showing the top three predicted off-target loci (OT-1, OT-2, and OT-3) following prime editing–mediated introduction of the PS1^{E280A} mutation into non-demented control (NDC) hiPSCs. Sequencing traces from parental NDC cells and PS1E280A-edited clones demonstrate identical nucleotide sequences across all analyzed off-target sites, indicating absence of detectable off-target editing events.

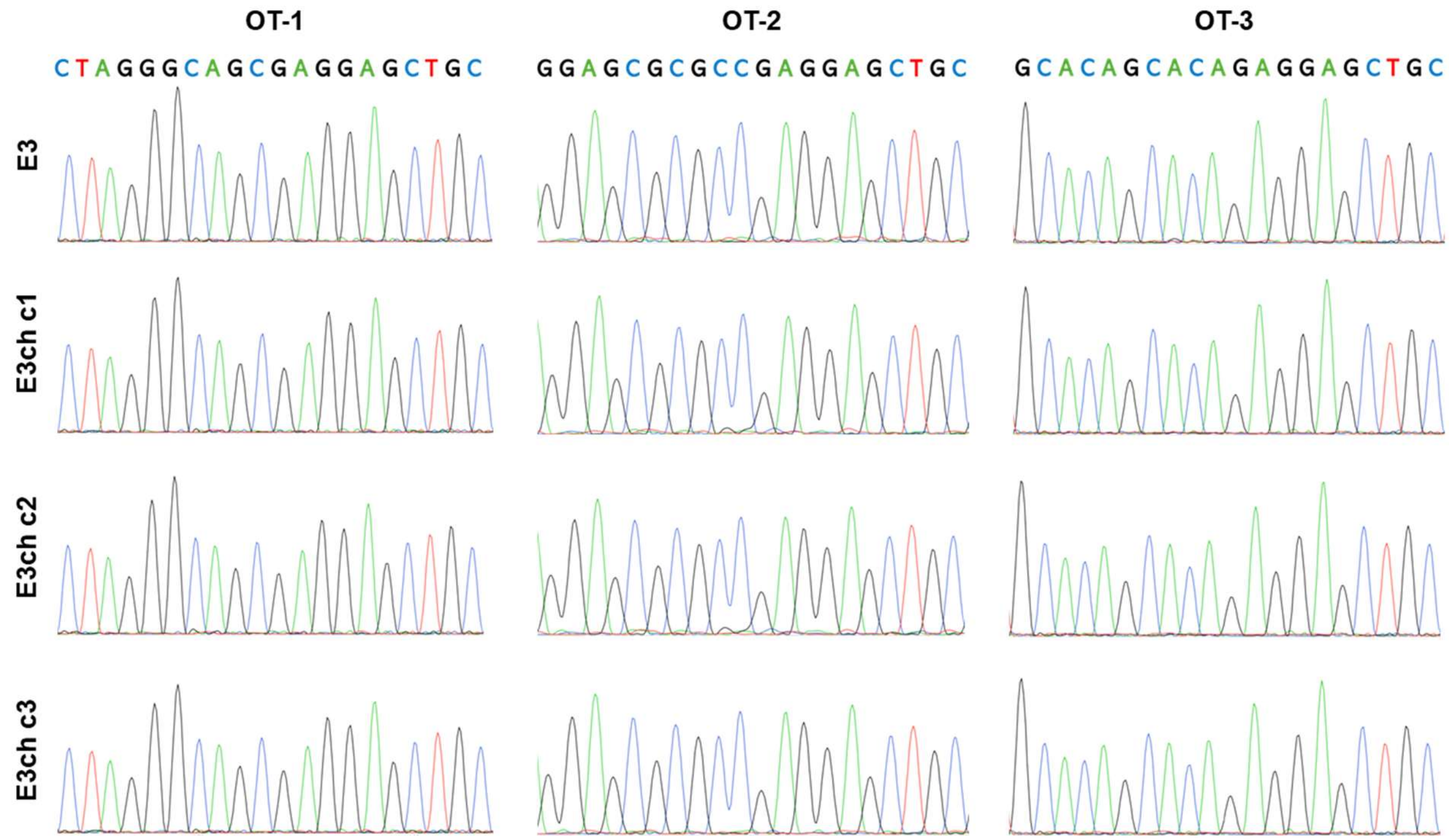

Supplemental Figure 2. Off-target analysis of APOE3ch prime editing in PS1^{E280A} hiPSC clones. Representative Sanger sequencing chromatograms of the top predicted off-target loci (OT-1, OT-2, and OT-3) following prime editing–mediated introduction of the APOE3 Christchurch (APOE3ch) mutation into PS1^{E280A} hiPSCs. Sequencing traces from parental PS1^{E280A} APOE3 cells and APOE3ch-edited clones demonstrate identical nucleotide sequences across all analyzed off-target sites, indicating absence of detectable off-target editing events.

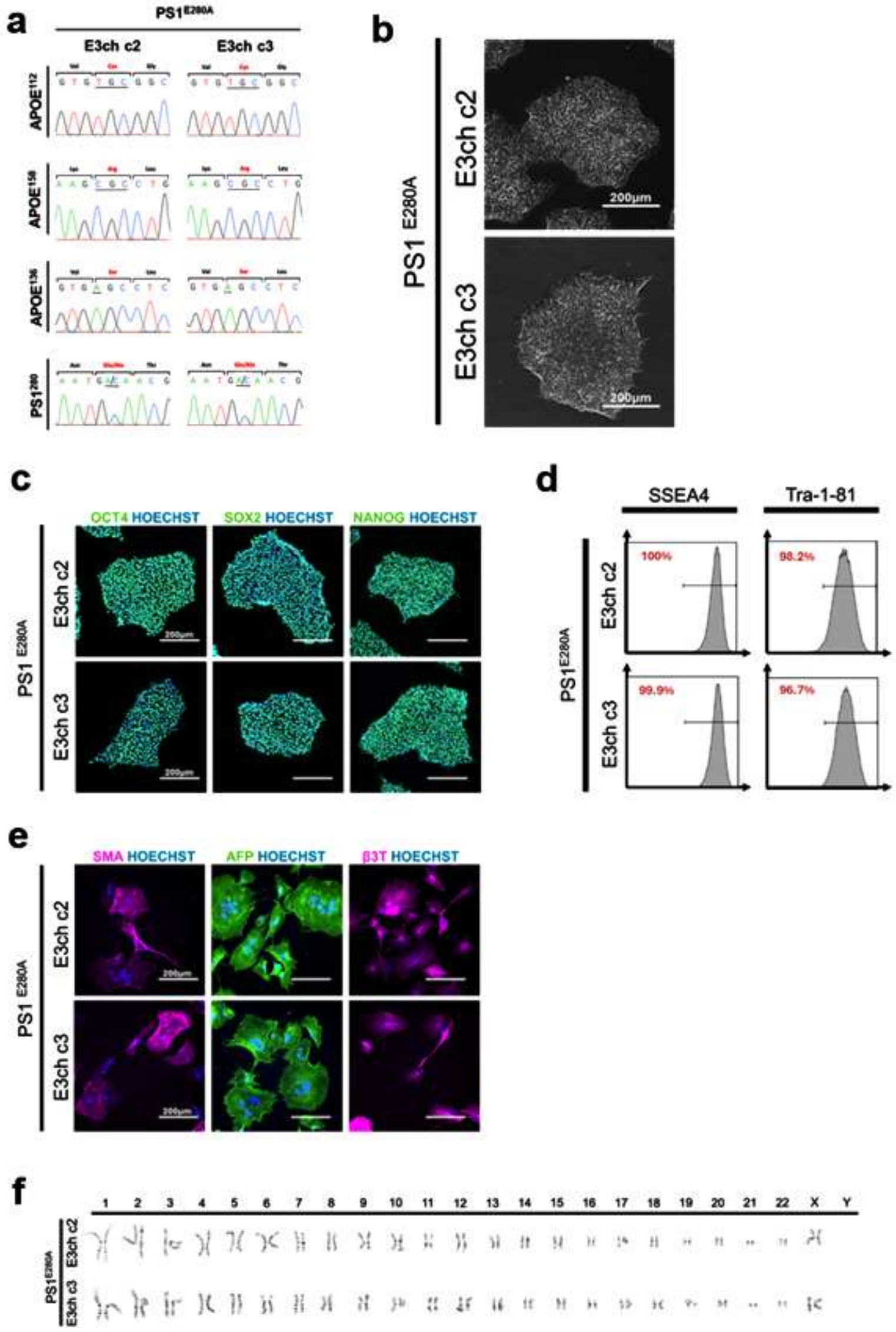

Supplemental Figure 3. Characterization of additional PS1^{E280A} APOE3ch isogenic hiPSC clones.(a) Sanger sequencing chromatograms confirming successful generation of homozygous APOE3 Christchurch (APOE3ch) clones in PS1^{E280A} hiPSC lines. Editing was validated at APOE codons 112, 136, and 158. (b) Representative phase-contrast images demonstrating typical hiPSC colony morphology across parental edited clones. (c) Immunofluorescence staining showing robust expression of pluripotency markers OCT4, SOX2, and NANOG across parental and edited hiPSC lines. (d) Flow cytometry analysis demonstrating high expression of pluripotency-associated surface markers TRA-1-81 and SSEA4 in APOE3ch clones. (e) Trilineage differentiation assays demonstrating retained differentiation potential into ectoderm, mesoderm, and endoderm lineages following genome editing. (f) G-banded karyotype analysis confirming normal euploid chromosomal integrity across all additional PS1^{E280A} APOE3ch clones.

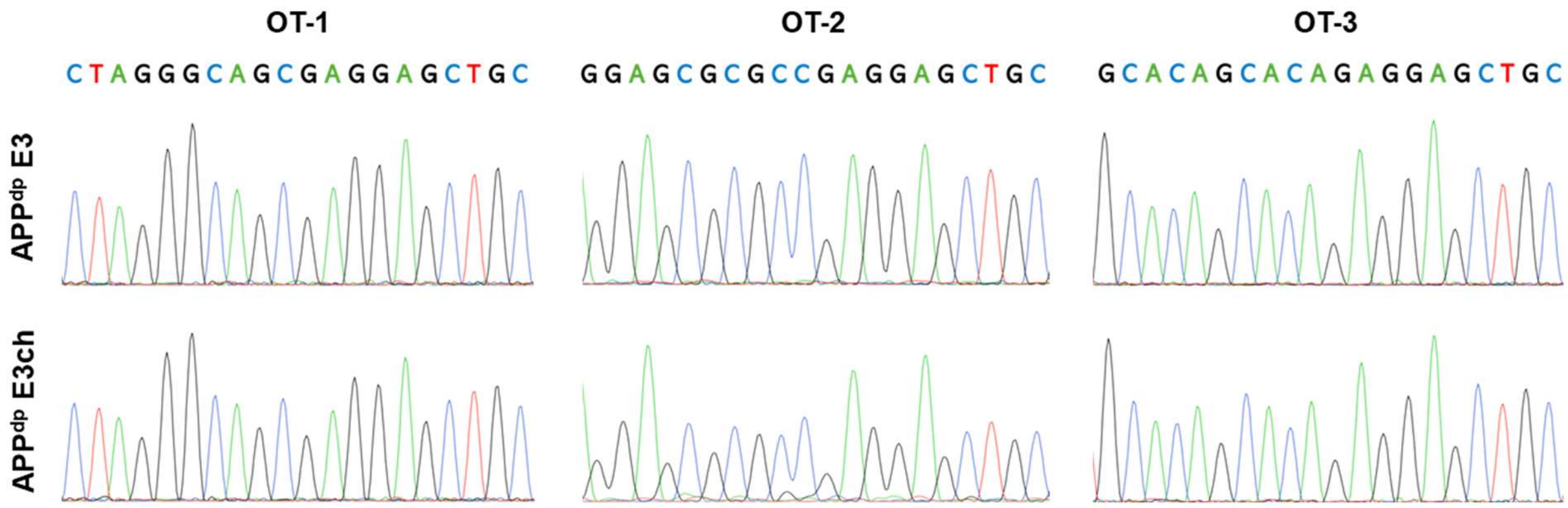

Supplemental Figure 4. Off-target analysis of APOE3ch prime editing in APP^{dp} hiPSC clones. Representative Sanger sequencing chromatograms of the top predicted off-target loci (OT-1, OT-2, and OT-3) following prime editing–mediated introduction of the APOE3 Christchurch (APOE3ch) mutation into APP^{dp} hiPSCs. Sequencing traces from parental APP^{dp} APOE3 cells and APOE3ch-edited clones demonstrate identical nucleotide sequences across all analyzed off-target sites, indicating absence of detectable off-target editing events.

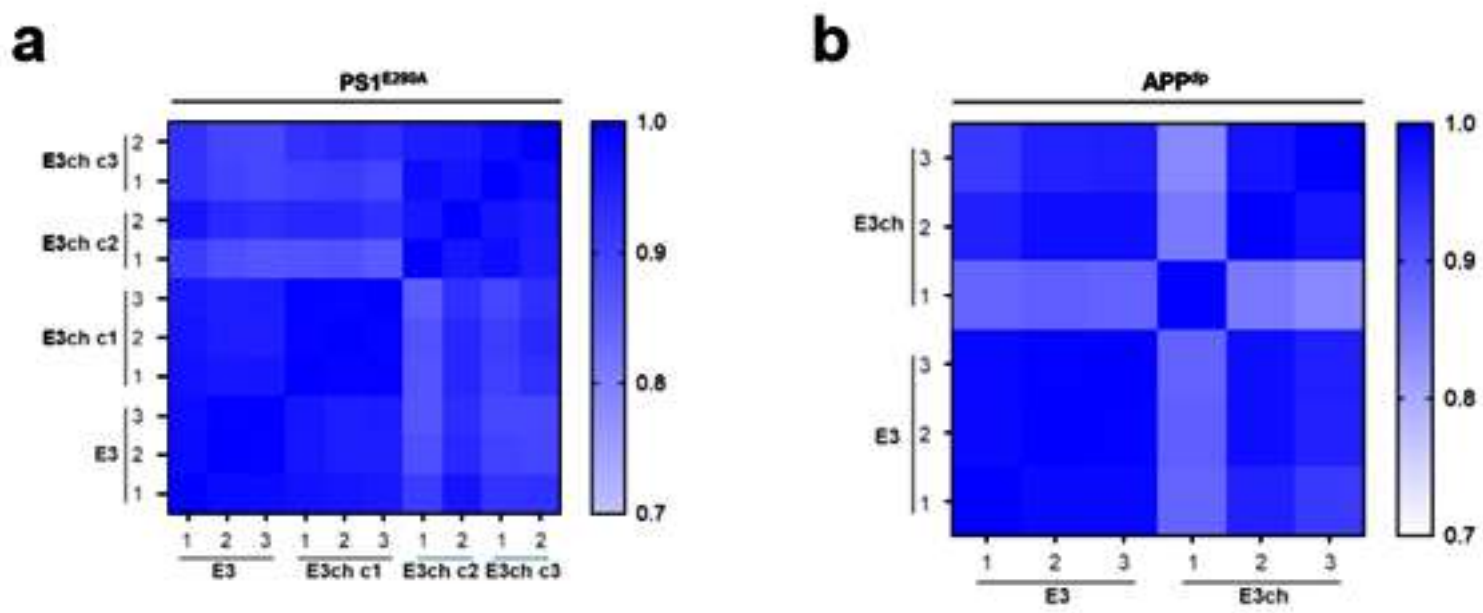

Supplemental Figure 5. Reproducibility of transcriptomic profiles across independent neural differentiations. (a) Global transcriptomic correlation analysis of RNA sequencing datasets from PS1^{E280A} APOE3 and APOE3ch neural cultures generated across independent differentiations. Correlation matrices demonstrate strong similarity between independent differentiations and clones, indicating high reproducibility of differentiation and gene expression patterns. (b) Global transcriptomic correlation analysis of RNA sequencing datasets from APP duplication APP^{dp} APOE3 and APOE3ch neural cultures generated across independent differentiations. Correlation matrices demonstrate strong similarity between independent differentiations and clones, indicating high reproducibility of differentiation and gene expression.

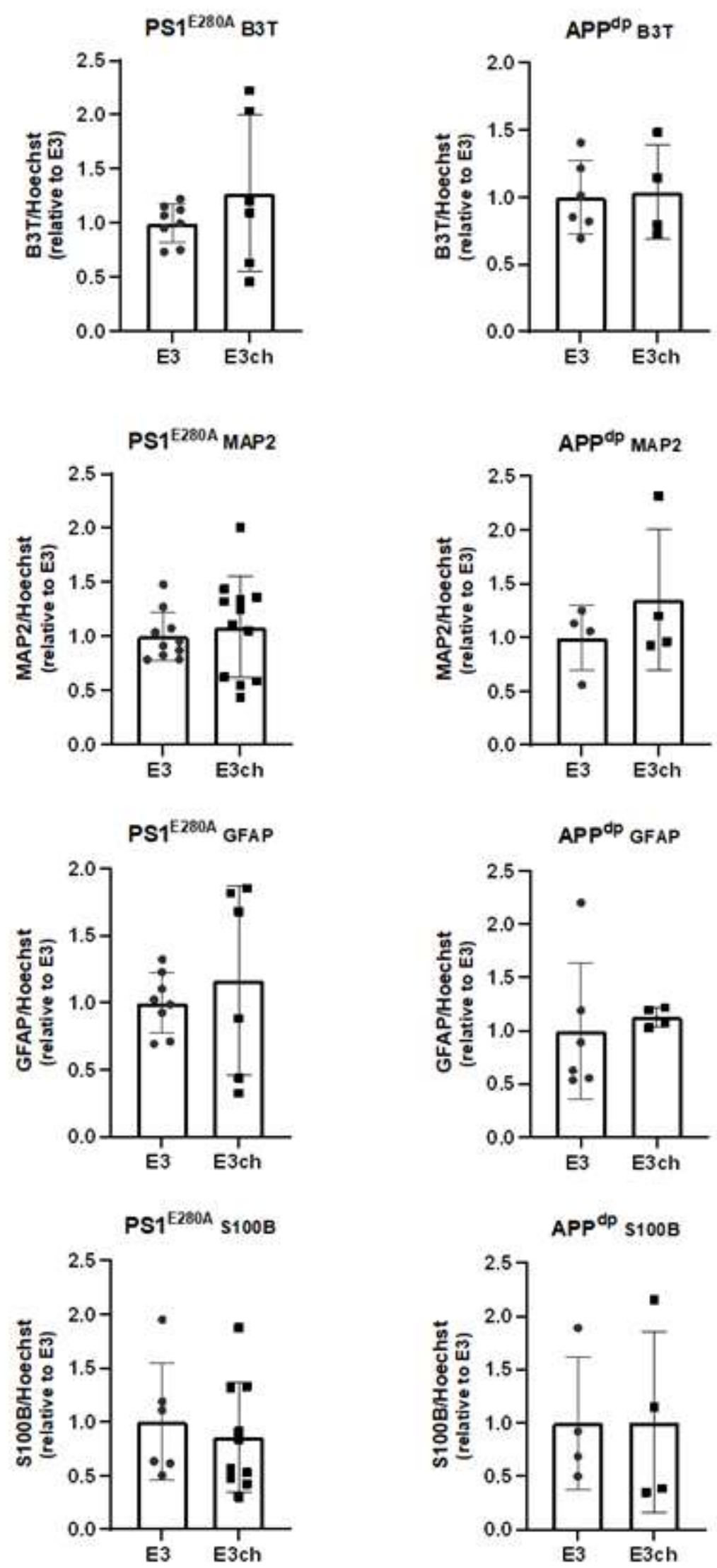

Supplemental Figure 6. Quantification of neuronal and astrocytic marker expression in PS1^{E280A} and APP^{dp} neural cultures. Quantification of immunofluorescence staining for the neuronal markers TUJ1 and MAP2 as well as the astrocytic markers S100β and GFAP across PS1^{E280A} and APP^{dp} APOE3 and APOE3ch neural cultures.

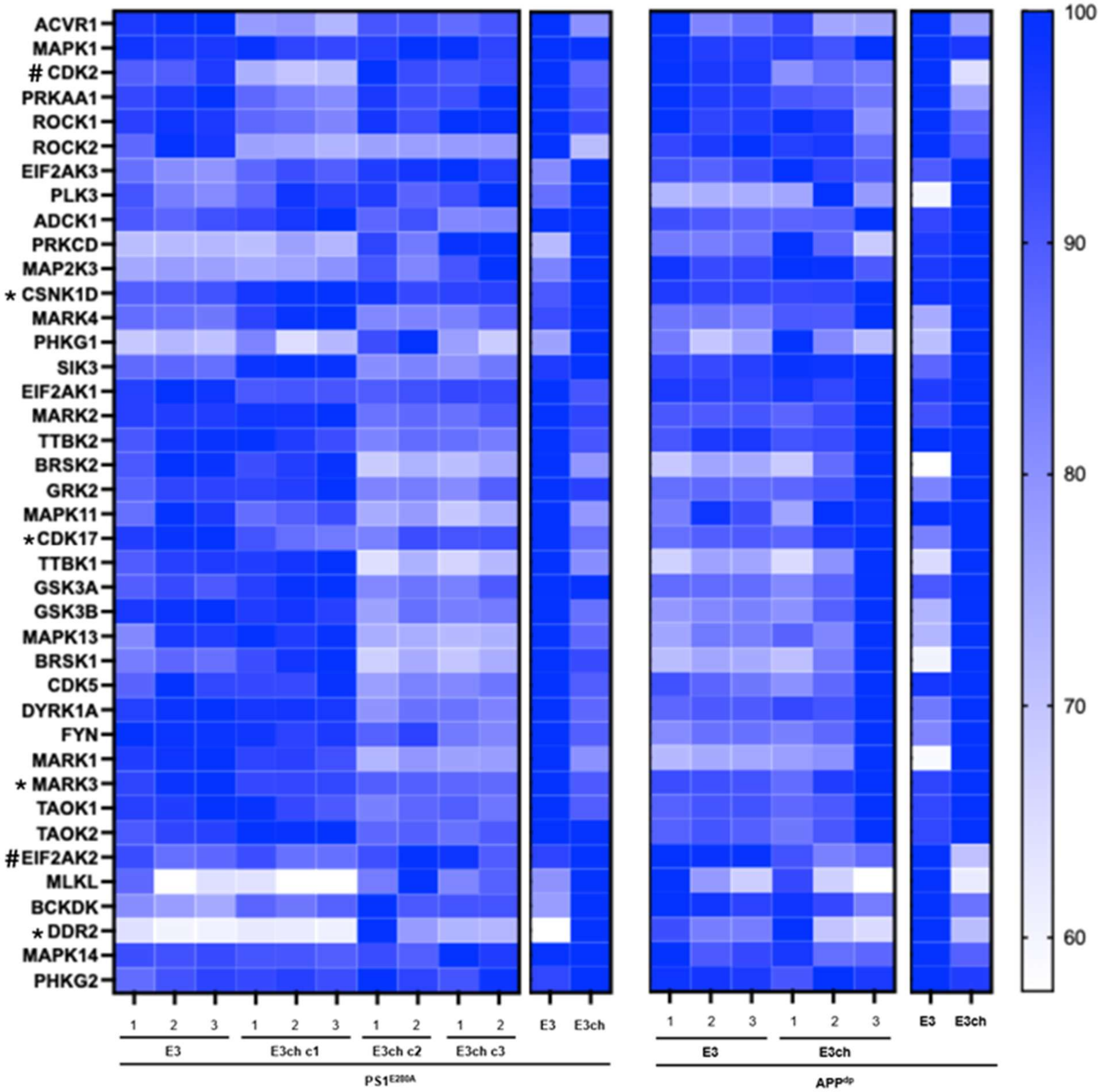

Supplemental Figure 7. Tau-associated kinase gene expression in PS1^{E280A} and APP^{dp} neural cultures. Heatmap of tau-associated kinase gene expression across PS1^{E280A} and APP^{dp} APOE3 and APOE3ch neural cultures. Significance in PSEN1 E280A cultures is denoted by an asterisk (*), and significance in APPdp cultures is denoted by a number sign (#). For RNA-seq data, each column represents an independent well from independent differentiations.
