# Supplementary material for: Isogenic human stem cell models reveal mutation-dependent protective mechanisms of APOE3 Christchurch
Source: Front Aging Neurosci. 2026 Oct 1;18:1856154. doi: 10.3389/fnagi.2026.1856154 (PMC13669193; doi:10.3389/fnagi.2026.1856154)
Supplement: Supplementary file 2 [file Data_Sheet_2.pdf]

Supplemental Table 1. PegRNA and nsgRNA sequences used in this study.

Target Site (guide system, pegRNA components)	pegRNA			nsgRNA	
	Spacer Sequence	Hairpin Sequence	3’ Extension Sequence	Spacer Sequence	Hairpin Sequence
BFP-to-GFP (PE3, pegRNA)	CTCGTGACCACCCTGACCA	GTTTTAGAGCTAGAAATAGCAAGTTAAAATAAGGCTAGTCCGTTATCAACTTGAAAAAGTGGCACCGAGTCGG	CACGCCGTAGGTCAGGGTGGTCA	GACGTAGCCTTCGGGCATGG	GTTTTAGAGCTAGAAATAGCAAGTTAAAATAAGGCTAGTCCGTTATCAACTTGAAAAAGTGGCACCGAGTCGG
APOE R136S (PE3b, epegRNA with linker sequence and **tevopreQ1 RNA motif**)	CCAGAGCACCGAGGAGCTGC	GTTTTAGAGCTAGAAATAGCAAGTTAAAATAAGGCTAGTCCGTTATCAACTTGAAAAAGTGGCACCGAGTCGG	GAGGCGAGGCTCACCCGCAGCTCCTCGGTGCTAGTAATGA**CGCGGTTCTATCTACTTACGCGTTAAACCAACTAGAA**	GAGGCTCACCCGCAGCTCCT	GTTTTAGAGCTAGAAATAGCAAGTTAAAATAAGGCTAGTCCGTTATCAACTTGAAAAAGTGGCACCGAGTCGG
PSEN1 E280A (PE3, epegRNA with **tevopreQ1 RNA motif**)	TATGCTGGTTGAAACAGCTC	GTTTTAGAGCTAGAAATAGCAAGTTAAAATAAGGCTAGTCCGTTATCAACTTGAAAAAGTGGCACCGAGTCGG	AAAAAGCGTTGCATTTCTCTCCTGAGCTGTTTCAACCAG**CGCGGTTCTATCTACTTACGCGTTAAACCAACTAGAA**	TATCCTTCTCAAATACTTAC	GTTTTAGAGCTAGAAATAGCAAGTTAAAATAAGGCTAGTCCGTTATCAACTTGAAAAAGTGGCACCGAGTCGG

Supplemental Table 2. List of primers used in this study.

Primer	Forward Sequence (5' →3')	Reverse Sequence (5' →3')
APOE R136S EXT	GGACGAGACCATGAAGGAGTTGAAGGC	CCACCTGCTCCTTCACCTCGTCCAG
APOE R136S INT	ACGGCTGTCCAAGGAGCTG	CCCCGGCCTGGTACACTG
PSEN1 E280A EXT	CCACCAGTTCACCTGCCATT	GGAATGCTGGTTCCCCAATA
PSEN1 E280A INT	TGGCTGTTTTGTGTCCGAAA	TCTGCAGGAGTTCCAGGAATG
APOE R136S OT-1	GGTGGAGGAAAGTGGTGGTG	TCTCCAAGCCACTGTGACTC
APOE R136S OT-2	CAGGCTCTGAGCTGGTTCTG	CTGACTTCAGAGGCCACACC
APOE R136S OT-3	GGGTCACCAAATAAGGGCCT	CTCTGCCCTCTCCCAGGT
PSEN1 E280A OT-1	GAGGCTCCCGTGGTCTGAGA	GGCCCAGGAACGAGAGAGGA
PSEN1 E280A OT-2	GCTGACATGAACTTAGTGGGACA	AGCTCCTCAGTGTGCGGGTA
PSEN1 E280A OT-3	GCCATGATCACACCACTGCAC	CCCCTCAAACATGATCCCAGG

Supplemental Table 3. List of antibodies used in this study.

**Antibody**	**Vendor**	**Catalog Number**	**Dilution**
Anti-human NANOG Rabbit pAb	ThermoFisher Scientific	Cat# PA1-097, RRID: AB_2539867	1:200
Anti-human OCT4 Rabbit pAb	ThermoFisher Scientific	Cat# PA5-27438, RRID: AB_2544914	1:200
Anti-human SOX2 Rabbit pAb	ThermoFisher Scientific	Cat# PA1-094, RRID: AB_2539862	1:200
Human/Mouse SSEA-4 APC-conjugated Antibody	R&D Systems	Cat# FAB1435A, RRID: AB_494994	1:10
Mouse IgG3 APC-conjugated Antibody	R&D Systems	Cat# IC007A, RRID: 952035	1:10
Alexa Fluor® 647 anti-human TRA-1-81 Antibody	BioLegend	Cat# 330705, RRID: AB_1089242	1:10
Alexa Fluor® 647 Mouse IgM, k Isotype Ctrl Antibody	BioLegend	Cat# 401618, RRID: AB_2802167	1:10
Smooth Muscle Actin Antibody (CGA7)	Santa Cruz Biotechnology	Cat# sc53015, RRID: AB_628683	1:50
AFP Polyclonal Antibody	ThermoFisher Scientific	Cat# PA5-21004, RRID: AB_11157055	1:50
Polyclonal Rabbit Anti-MAP2 antibody	MilliporeSigma	Cat# AB5622, RRID: AB_91939	1:500
Anti-beta III Tubulin antibody [2G10] – Neuronal Marker	Abcam	Cat# ab78078, RRID: AB_2256751	1:1000
Anti-Neun, clone A60	EMD Milliore	Cat# MAB377, RRID: AB_2298772	1:500
Anti-S100B antibody		Cat# S2532, RRID: AB_477499	1:500
Donkey Anti-rabbit Alexa 488 IgG (H+L)	ThermoFisher Scientific	Cat# A-21206, RRID: AB_2535792	1:200

Donkey anti-mouse Alexa 488 IgG (H+L)	ThermoFisher Scientific	Cat# A21202,<br>RRID: AB_141607	1:200
Donkey anti-Mouse IgG (H+L) Alexa Fluor 647	ThermoFisher Scientific	Cat# A21571,<br>RRID: AB_162542	1:200
